# Dual Roles of Natural Products in Regulating Ferroptosis in Acute and Chronic Liver Diseases: A Review

**DOI:** 10.1155/omcl/9874221

**Published:** 2026-08-18

**Authors:** Farshad Niazpour, Reza Meshkani

**Affiliations:** ^1^ The Ritchie Centre, Hudson Institute of Medical Research, Melbourne, Australia, hudson.org.au; ^2^ Department of Paediatrics, Monash University, Melbourne, Australia, monash.ac.za; ^3^ Department of Clinical Biochemistry, School of Medicine, Tehran University of Medical Sciences, Tehran, Iran, tums.ac.ir

**Keywords:** ferroptosis, HCC, liver diseases, liver fibrosis, NAFLD, natural products

## Abstract

Liver diseases, both acute and chronic, are a major global health burden with limited therapeutic options. Ferroptosis, an iron‐dependent form of regulated cell death driven by lipid peroxidation, has emerged as a key contributor to liver pathology and a potential therapeutic target. This review examines how plant‐derived natural products modulate ferroptosis across acute liver failure (ALF), nonalcoholic fatty liver disease (NAFLD), nonalcoholic steatohepatitis (NASH), liver fibrosis, and hepatocellular carcinoma (HCC). These compounds show dual effects, suppressing ferroptosis to protect hepatocytes in ALF, NAFLD, and NASH, and inducing ferroptosis to eliminate activated hepatic stellate cells and cancer cells in liver fibrosis and HCC. Mechanistically, they act on major ferroptosis regulators, including nuclear factor erythroid 2‐related factor 2 (NRF2), solute carrier family 7 member 11 (SLC7A11), glutathione (GSH) peroxidase 4 (GPX4), heme oxygenase 1 (HMOX1), acyl‐CoA synthetase long‐chain family member 4 (ACSL4), lipid metabolism enzymes including arachidonate lipoxygenases (ALOXs) and lysophosphatidylcholine acyltransferase 3 (LPCAT3), and tumor protein p53 (p53). They influence transcriptional and epigenetic programs to regulate gene expression related to ferroptosis. Some natural products modulate noncoding RNAs and chromatin‐modifying enzymes to suppress genes promoting ferroptosis, while others affect RNA methylation to stabilize antioxidant defenses. Additionally, these compounds regulate iron metabolism and impact key upstream signaling pathways, collectively shaping ferroptosis control. Despite promising preclinical results, translation to clinical use is challenged by context‐dependent effects, bioavailability limitations, and potential toxicity. Further mechanistic studies, in vivo validation, and clinical trials are warranted. Overall, natural product‐based modulation of ferroptosis offers a promising therapeutic avenue in liver disease.

## 1. Introduction

Liver diseases, including both acute and chronic forms (defined as lasting over 6 months), impair the liver’s vital functions. Acute liver diseases arise due to a substantial loss of functional liver tissue following hepatocyte death triggered by acute injury. They are commonly caused by agents such as hepatitis B and C viruses, excessive alcohol consumption, environmental toxins, and certain drugs that damage hepatocytes, resulting in hepatotoxicity. Their incidence varies significantly across different regions. In developed nations, the annual occurrence is estimated to range between 1 and 6 cases per million people. In developing countries, largely due to the absence of widespread vaccination programs against hepatitis viruses, it is more widespread [1].

Chronic liver diseases typically arise from metabolic disturbances. Among these, nonalcoholic fatty liver disease (NAFLD) is currently the most prevalent chronic liver disease. NAFLD is characterized by the accumulation of lipids (steatosis) in the liver that occurs independently of excessive alcohol intake. Globally, ~25%–30% of the population is affected by NAFLD. Although NAFLD is often considered a relatively nonprogressive condition with minimal health risks, it can progress to a more severe form known as nonalcoholic steatohepatitis (NASH). Studies indicate that around 20%–30% of individuals with NAFLD will develop NASH if not properly managed. NASH is characterized by pronounced steatosis, along with hepatocyte ballooning and inflammation. NASH is estimated to affect 3%–5% of the global population, and ~40% of individuals with NASH may go on to develop fibrosis and cirrhosis if left uncontrolled. Currently, no approved pharmacological treatments exist for either NAFLD or NASH, and lifestyle interventions such as weight loss and dietary modifications remain the primary strategies to halt disease progression [2–4].

Liver fibrosis is a key feature of chronic liver disease, marked by ongoing inflammation and tissue damage that progressively impair liver function. Early detection of fibrosis stages is crucial as advancing fibrosis significantly increases the risk of developing cirrhosis, liver failure, and hepatocellular carcinoma (HCC). The prevalence of liver fibrosis varies globally, influenced by regional risk factors such as obesity, alcohol use, and viral hepatitis infections. The activation of hepatic stellate cells (HSCs) is a central feature of liver fibrosis, and targeting this process offers promising therapeutic potential. However, once fibrosis progresses to cirrhosis, treatment options become limited, and in severe cases, liver transplantation may be the only viable option [5].

HCC represents the most severe and advanced stage of chronic liver disease. It is the sixth most commonly diagnosed cancer worldwide, yet despite improvements in diagnosis and treatment, the prognosis for HCC remains poor. Traditionally, curative options have been limited to surgical resection and liver transplantation. However, recent advancements have expanded treatment modalities to include minimally invasive interventions, immunotherapies, and combination therapies. Nonetheless, current treatments are still inadequate for many patients, highlighting the urgent need for more effective and lasting therapeutic strategies to enhance long‐term survival [6].

As noted, liver diseases are widespread and impose significant long‐term health challenges, often with few effective treatment options available. These limitations have driven ongoing efforts to better understand the underlying mechanisms involved in their pathogenesis. Among the emerging areas of research are ferroptosis and a unique form of cell death. Since its discovery, ferroptosis has gained substantial interest for its involvement in the onset and progression of a wide range of diseases, such as ischemia‐reperfusion injury, inflammatory bowel disease, diabetic kidney disease, Alzheimer’s disease, cardiovascular and immune‐related diseases, various cancers, and liver diseases [7].

A variety of pharmacological agents have been shown to influence ferroptosis. Thus, ferroptosis modulation‐based therapies seem to be effective in liver diseases. Although these agents serve as important tools for studying ferroptosis, many are associated with notable side effects, limiting their current clinical use. In contrast, a growing body of evidence suggests that naturally occurring plant‐derived compounds—commonly referred to as natural products—have demonstrated the ability to modulate ferroptosis‐related pathways with fewer side effects across a wide range of diseases [8, 9].

This review addresses critical gaps in the current literature by providing a systematic, mechanistic analysis of how natural products regulate ferroptosis across the full spectrum of liver diseases, spanning acute injuries, chronic conditions, and HCC. While several reviews have touched on related topics, they remain limited in scope and fail to address key nuances. Hemmati et al. [10] examined ferroptosis modulation in hepatotoxicity but focused broadly on mesenchymal stem cell derivatives, nanoparticle systems, and natural products without distinguishing disease‐specific mechanisms or providing comprehensive coverage of natural products alone. Yang et al. [11] demonstrated ferroptosis modulation by traditional Chinese medicine in liver cancer; however, their scope was restricted to traditional Chinese medicine, excluded acute liver diseases and nonmalignant chronic conditions, and covered publications from 2021 to 2025, leaving earlier mechanistic insights unaddressed.

Existing reviews treat acute and chronic liver diseases separately without systematic comparison of how the same natural products may exert opposite therapeutic effects depending on the disease stage. We argue that a comprehensive, disease‐stage‐specific review is essential because liver diseases are progressive. For instance, NAFLD can evolve into chronic fibrosis, cirrhosis, and ultimately, HCC [12]. Therefore, understanding ferroptosis regulation at each disease stage is critical for developing stage‐specific therapeutic strategies.

In addition, natural products exhibit dualistic, context‐dependent effects. A single compound may inhibit ferroptosis in acute liver injury (therapeutic) but induce ferroptosis in HCC cells (also therapeutic). This paradoxical duality remains unsystematized in the current literature. Therefore, a mechanistic integration is required that captures all mechanisms simultaneously to enable researchers to overcome the current challenges in natural product development and clinical translation.

This review distinguishes the nuanced regulatory mechanisms of natural products across acute and chronic liver conditions to elucidate their context‐dependent effects, illustrating how a single compound can either induce or inhibit ferroptosis depending on the pathology. Ultimately, we aim to identify key therapeutic targets and provide mechanistic insights that serve as a comprehensive guide for future translational studies.

## 2. Overview of Ferroptosis

### 2.1. Morphological and Biochemical Features

Ferroptosis is a distinct form of regulated cell death first identified in 2012 during studies on selective toxicity in RAS‐mutant cells. Unlike classical cell death pathways such as apoptosis, necrosis, and autophagy, ferroptosis is uniquely characterized by its iron dependency, depletion of glutathione (GSH), and accumulation of lipid reactive oxygen species (ROS) [13, 14].

Morphologically and biochemically, ferroptosis differs markedly from other cell‐death modalities. Apoptosis maintains plasma membrane integrity and forms apoptotic bodies, while necrosis involves cell swelling, nuclear condensation and fragmentation, and the rupture of organelles. Autophagy is marked by autophagosome formation and cytoplasmic degradation. In contrast, ferroptosis is distinguished by characteristic mitochondrial changes, including shrinkage, increased membrane density, loss or reduction of cristae, and membrane vesiculation, ultimately leading to plasma membrane rupture without chromatin condensation [15, 16].

### 2.2. Mechanisms and Mediators of Ferroptosis

Ferroptosis is driven by lethal lipid peroxidation through a complex interplay of enzymatic and nonenzymatic processes that damage membrane integrity and lead to cell death. Lipid peroxidation results from the reaction of unsaturated fatty acids with oxygen, forming reactive lipid radicals, such as lipid peroxides and hydroperoxides. These radicals initiate chain reactions that propagate further lipid peroxidation, inducing oxidative damage. Toxic breakdown products like malondialdehyde (MDA) and 4‐hydroxynonenal (4‐HNE) accumulate, damaging DNA and proteins and exacerbating cellular injury. Persistent lipid peroxidation compromises the lipid bilayer’s structural integrity, impairing cell viability [17].

As stated, ferroptosis execution involves both enzymatic and nonenzymatic processes. Enzymatically, oxidation is catalyzed by arachidonate lipoxygenases (ALOXs), iron‐containing enzymes that oxidize polyunsaturated fatty acids such as arachidonic acid and adrenic acid. The synthesis of ferroptosis‐related lipid substrates involves acyl‐CoA synthetase long chain family member 4 (ACSL4), which converts free arachidonic acid and adrenic acid into CoA derivatives, followed by lysophosphatidylcholine acyltransferase 3 (LPCAT3)‐mediated esterification into membrane phospholipids like phosphatidylethanolamine. The accumulation of oxidized phospholipid hydroperoxides destabilizes membranes and triggers ferroptosis [18, 19].

Nonenzymatically, ferroptosis is driven by iron‐dependent reactions that intensify lipid peroxidation. Dysregulated iron metabolism increases cytosolic ferrous iron (Fe^2+^), which catalyzes the Fenton reaction with hydrogen peroxide, generating highly reactive hydroxyl radicals. These radicals abstract hydrogen atoms from membrane lipids, initiating lipid peroxidation and exacerbating oxidative damage. Iron homeostasis is tightly regulated by transferrin, transferrin receptor 1 (TFR1), ferroportin (FPN), and ferritin, which control iron uptake, storage, and export. Dysfunction or iron overload expands the labile iron pool (LIP), enhancing Fenton chemistry and promoting ferroptosis [20].

Ferroptosis is accompanied by severe mitochondrial alterations, highlighting the organelle’s central role in this iron‐dependent death. Mitochondria, rich in iron and major sources of ROS, contribute to ferroptosis by disrupting the iron balance within the organelle. This imbalance increases oxidative stress and drives lipid peroxidation, which damages proteins, lipids, DNA, and carbohydrates, altering mitochondrial function and dynamics. ROS, including superoxide, hydrogen peroxide, hydroxyl radicals, and lipid peroxides, are primarily generated by nicotinamide adenine dinucleotide phosphate (NADPH) oxidases and the mitochondrial electron transport chain. Their elevated presence amplifies ferroptosis signaling, while mitochondria‐targeted antioxidants can suppress ferroptotic death, underscoring mitochondria’s pivotal regulatory role [21].

Central to ferroptosis regulation is the GSH peroxidase 4 (GPX4) axis. GPX4 reduces toxic lipid hydroperoxides to inert lipid alcohols, preserving membrane integrity. Its activity depends on GSH synthesized from glutamate, glycine, and cysteine. Cysteine, the rate‐limiting substrate, is imported primarily via System Xc^−^, a membrane antiporter composed of solute carrier family 7 member 11 (SLC7A11) (light chain) and solute carrier family 3 member 2 (SLC3A2) (heavy chain) subunits, exchanging extracellular cystine for intracellular glutamate. Downregulation of SLC7A11 reduces cystine uptake, depletes GSH, and sensitizes cells to ferroptosis, whereas upregulation enhances antioxidant defenses and may contribute to tumor chemoresistance [22].

Beyond GPX4, ferroptosis suppressor protein 1 (FSP1) offers a parallel protective mechanism by reducing Coenzyme Q10 (CoQ10) to its antioxidant form ubiquinol using NADPH, scavenging lipid peroxyl radicals independently of GSH, and safeguarding cells when GPX4 is impaired [23].

Nuclear factor erythroid 2‐related factor 2 (NRF2) is another key ferroptosis inhibitor. This transcription factor orchestrates cellular defenses against oxidative stress by regulating antioxidant enzyme production. Normally restrained by kelch‐like ECH‐associated protein 1 (KEAP1), oxidative stress induces NRF2 release, allowing its nuclear translocation to activate genes involved in antioxidant defense, GSH synthesis, SLC7A11 expression, and iron metabolism. By upregulating antioxidants such as GPX4, NRF2 neutralizes lipid radicals and prevents ferroptosis‐associated damage. It also controls iron uptake, storage, and release, reducing intracellular iron accumulation and maintaining a redox balance to protect cells [24]. One critical downstream target of NRF2 is heme oxygenase‐1 (HMOX1), which degrades heme into ferrous iron; while generally antioxidant, HMOX1 can promote ferroptosis under certain conditions by increasing intracellular iron levels [25]. Conversely, the tumor suppressor p53 facilitates ferroptosis by repressing SLC7A11 expression, enhancing iron uptake and metabolism, and increasing ROS production [26].

In summary, ferroptosis is a tightly regulated form of cell death driven by the interplay between the enzymatic oxidation of polyunsaturated fatty acids and iron‐catalyzed nonenzymatic lipid peroxidation. Its execution relies on mitochondrial iron metabolism, ROS generation, and lipid membrane destabilization. Cellular antioxidant systems, including the GSH/GPX4 axis, System Xc^−^‐mediated cystine import, KEAP1/NRF2/antioxidant genes, and alternative pathways such as FSP1/CoQ10, work together to prevent ferroptosis. Disruption of this delicate balance leads to ferroptotic cell death, highlighting ferroptosis as a promising therapeutic target for diseases.

## 3. Acute Liver Failure (ALF)

ALF is defined as severe acute liver injury with hepatic encephalopathy and impaired synthetic function in individuals without pre‐existing chronic liver disease. The most well‐known clinical manifestations of the disease include jaundice, ascites, and hepatic encephalopathy [27]. The major causes of ALF include viral infections, drug‐induced toxicity, chronic alcohol consumption, ischemia, and other less common etiologies [28].

These pathogenic factors result in damage to hepatocytes and also Kupffer cells, which normally protect hepatocytes from injuries, and can cause apoptosis and necrosis in the hepatocytes, ultimately predisposing the tissue to inflammation. Collectively, these damages expand and lead to a marked loss in hepatic cells, exceeding the liver’s capacity for regeneration. These structural and immunological alterations can trigger systemic signaling to other organs, thereby increasing susceptibility to multi‐organ failure [29].

In developing countries, poor sanitation and limited vaccination coverage make viral infections the leading cause of ALF, particularly hepatitis A and hepatitis E. According to Patterson et al. [30], vaccination programs have led to a marked reduction in the prevalence of virus‐induced ALF. For instance, immunization initiatives in Argentina reduced the proportion of virally induced ALF cases from 50% to 1%. Despite these advances, ALF remains associated with high mortality rates, ranging from 12% to 40% in high‐income countries and from 18% to 91% in lower‐middle‐income countries. Overall, this condition represents a significant global health burden, underscoring the need for well‐planned and effective strategies for both the prevention and treatment of ALF.

In developed countries, the primary cause of hepatotoxicity is drug‐ and toxin‐induced ALF, regardless of the specific substance involved. Most cases of drug‐induced ALF are diagnosed with acetaminophen intoxication, and this type of intoxication is particularly dangerous in individuals with a history of alcohol consumption as the liver becomes more susceptible to injury. Drug‐induced ALF leads to mitochondrial dysfunction, oxidative stress, and apoptosis or necroptosis of hepatocytes, ultimately promoting the spread of inflammation. Elevated liver enzymes, including alanine aminotransferase (ALT), aspartate aminotransferase (AST), and alkaline phosphatase (ALP), are key laboratory findings in drug‐induced ALF. Although drug withdrawal leads to significant improvement, liver transplantation may be required in severe cases, highlighting the need for further mechanistic studies into the pathogenesis of the disease [31].

Alcoholic liver disease (ALD), which results from prolonged alcohol consumption, represents another major cause of ALF. It is characterized by a multifaceted pathophysiology involving hepatic steatosis, inflammation, oxidative stress, hepatocellular injury, neutrophil infiltration, dysregulated lipogenesis, adipocyte death, and the accumulation of toxic alcohol metabolites, all of which contribute to liver dysfunction [32]. Laboratory evaluation includes liver function tests, such as elevated levels of aminotransferases and the AST/ALT ratio, which assist in distinguishing ALD from other liver diseases. However, the diagnosis of ALD remains challenging until the disease reaches advanced stages as the early symptoms are often nonspecific. Despite extensive research into the pathogenesis of ALD, treatment options remain limited to alcohol cessation and liver transplantation in severe cases [33].

As stated, liver transplantation remains the only effective treatment for ALF severe cases, and the scientific community continues to call for more detailed mechanistic studies to identify novel signaling molecules involved in the pathogenesis of ALF. Notably, recent studies have underscored the involvement of ferroptosis in the progression of ALF, suggesting that it may serve as a key mechanistic driver of liver injury. Zhou et al. [34] demonstrated that ferroptosis was the main form of cell death in ALF, and that inhibiting components of the ferroptosis pathway protected mice against ALF induced by lipopolysaccharide/D‐galactosamine (LPS/D‐GalN).

Additionally, hepatitis B virus infection, one of the major causes of ALF, exacerbates D‐GalN‐induced liver injury by suppressing SLC7A11 expression, and treatment with a ferroptosis inhibitor mitigates this liver injury, supporting the role of ferroptosis in ALF [35].

Also, inhibition of ferroptosis using ferrostatin‐1 (Fer‐1) or α‐tocopherol attenuates liver damage in a murine model of hepatic ischemia/reperfusion injury, reinforcing the role of ferroptosis in the pathogenesis of ALF [36].

In conclusion, accumulating evidence from experimental models indicates that ferroptosis plays a significant role in the pathogenesis of ALF and that its modulation may yield promising therapeutic outcomes for patients. In recent years, several small molecules and pharmacological drugs have been shown to regulate ferroptosis mediators. Ferroptosis induction has been demonstrated to inhibit tumor growth and enhance tumor immunotherapy approaches [37].

In addition, natural products have been shown to regulate ferroptosis‐associated mechanisms and are of particular interest compared to synthetic compounds. Natural products possess structural complexity and diversity that synthetic compounds often cannot replicate, making them valuable for drug discovery. Their complex molecular structures provide multiple binding sites for protein targets, facilitating better interactions. Furthermore, from a historical perspective, natural products have been widely used in traditional medicine, conferring positive biological effects that confirm their biosafety and established safety profiles [38]. Although there are challenges regarding the use of natural products in clinical settings, including bioavailability, accessibility, side effects, and dose optimization, they remain of great interest. More research is easing the way to implement them in drug discovery.

The following section summarizes the mechanistic evidence demonstrating how natural products regulate ferroptosis in ALF, providing a foundation for understanding their therapeutic potential in this context.

### 3.1. Phenolic Compounds

Phenolic compounds are a diverse group of bioactive metabolites naturally found in plants and are broadly classified into major families, including polyphenols, flavonoids, lignans, stilbenes, and phenolic acids. These compounds contain one or more aromatic ring structures and have been widely investigated for their antioxidant, anti‐inflammatory, anticancer, anti‐aging, and cytoprotective properties [39].

Increasing evidence suggests that phenolic compounds can attenuate ALF through the modulation of ferroptosis. Various experimental models of ALF, including alcohol‐, drug‐, toxin‐, stress‐, and sepsis‐induced liver injury, have demonstrated the therapeutic potential of phenolic compounds in suppressing ferroptotic signaling and improving hepatic function.

Chronic restraint stress is known to induce liver injury through oxidative stress and ferroptosis. Chlorogenic acid, a naturally occurring polyphenol found in green coffee beans, protected rats against chronic stress‐induced liver injury. Chronic stress increased serum transaminase levels, ROS accumulation, iron overload, and ferroptosis, whereas chlorogenic acid treatment reversed these alterations and inhibited ferroptosis and mitochondrial apoptosis through activation of NRF2 signaling [40].

Excessive alcohol consumption can also lead to ALF, and several phenolic compounds have demonstrated protective effects against alcohol‐induced ferroptosis. Quercetin attenuated ethanol‐induced liver injury by suppressing ferroptosis, which was associated with reduced formation of mitochondria‐associated endoplasmic reticulum (ER) membranes and decreased expression of protein kinase R‐like endoplasmic reticulum (ER) kinase [41]. Similarly, flavonoid glycosides isolated from Abrus cantoniensis, particularly AH‐15, alleviated ALD through the inhibition of ferroptosis. Mechanistically, AH‐15 activated the AMP‐activated protein kinase (AMPK)/NRF2 pathway, thereby reducing oxidative stress and ferroptotic damage [42].

Acetaminophen overdose is one of the most common causes of drug‐induced ALF and has been strongly linked to ferroptosis‐mediated hepatocyte death. Several phenolic compounds have demonstrated hepatoprotective effects in acetaminophen‐induced liver injury models, including catechin, 7,8‐dihydroxyflavone, sesamin, scutellarin, sakuranetin, kaempferol, and rosmarinic acid. Catechin reduced serum liver enzymes, ROS generation, MDA accumulation, and GSH depletion in both in vitro and in vivo models. These effects were mediated through activation of the SLC7A11/GPX4 pathway, highlighting catechin as a natural ferroptosis inhibitor [43].

Consistent with these findings, 7,8‐dihydroxyflavone reduced oxidative stress and liver damage in a murine acetaminophen overdose model while restoring hepatic integrity and suppressing ferroptosis through upregulation of NRF2, GPX4, and SLC7A11. Importantly, inhibition of NRF2 abolished the protective effects of 7,8‐dihydroxyflavone, confirming the central role of NRF2 signaling in mediating ferroptosis suppression [44]. Sesamin also alleviated acetaminophen‐induced liver injury through modulation of ferroptosis‐, autophagy‐, and Forkhead box protein O1 (FOXO1)‐related pathways [45].

Likewise, scutellarin protected mice from acetaminophen‐induced hepatotoxicity by suppressing oxidative stress, apoptosis, pyroptosis, and ferroptosis. Its hepatoprotective effects depended on activation of the NRF2/SLC7A11/GPX4 axis, as these effects were abolished in Nrf2‐knockout mice [46]. Sakuranetin, a flavonoid isolated from cherry plants, also attenuated acetaminophen‐induced liver injury through the activation of NRF2 signaling and suppression of ferroptosis. Treatment reduced ALT and AST levels, inhibited ROS production, restored intracellular GSH, increased GPX4 and SLC7A11 expression, and downregulated ACSL4 [47].

Furthermore, kaempferol exhibited protective effects against acetaminophen‐induced liver injury by suppressing ferroptosis through NRF2 activation. Kaempferol reduced oxidative stress, iron accumulation, and mitochondrial ROS production while enhancing GPX4 expression and lipid peroxide detoxification. Molecular docking analyses additionally suggested that kaempferol may interact with KEAP1, thereby facilitating NRF2 activation [48].

Similarly, rosmarinic acid, a polyphenolic compound, mitigated acetaminophen‐induced liver injury in mice through the regulation of important pathways in ferroptosis. It was shown that rosmarinic acid improves liver function parameters, enhances antioxidant capacity, and inhibits ferroptosis through regulation of the SLC7A11/GPX4 pathway [49].

Carbon tetrachloride (CCl_4_) is a well‐established hepatotoxic agent commonly used to induce experimental ALF. Several phenolic compounds, including luteolin, schaftoside, curcumin, and total flavonoids extracted from Astragali Complanati Semen, have demonstrated protective effects against CCl_4_‐induced liver injury. Luteolin improved liver function by reducing serum AST, ALT, and ALP levels while suppressing ferroptosis through increased GPX4 expression, enhanced GSH levels, and reduced Fe^2+^ and MDA accumulation. SLC7A11 was identified as a key mediator of its protective effects in HepG2 cells [50].

Similarly, schaftoside, a bioactive compound isolated from *Clinacanthus nutans*, inhibited ferroptosis through activation of the NRF2/GPX4 pathway. Schaftoside reduced oxidative stress, reversed mitochondrial damage, and enhanced NRF2‐mediated GPX4 activation [51].

Curcumin also protected HepG2 cells against CCl_4_‐induced ALF by reducing ROS production, thioredoxin‐interacting protein (TXNIP) expression, NLRP3 inflammasome activation, and TFR1 expression. These effects were associated with the modulation of the TXNIP/NLRP3 pathway and the SLC7A11/GSH/GPX4 axis [52].

In addition, flavonoids extracted from Astragali Complanati Semen exhibited potent hepatoprotective effects against CCl_4_‐induced injury by reducing oxidative stress and iron accumulation, preserving mitochondrial ultrastructure, and suppressing ferroptosis‐related proteins such as TFR1 and voltage‐dependent anion channel 3 (VDAC3) [53].

Several phenolic compounds have also shown beneficial effects in sepsis‐ or LPS/D‐GalN‐induced ALF models. Isoferulic acid (3‐hydroxy‐4‐methoxycinnamic acid), a natural phenolic acid, alleviated sepsis‐induced liver injury in mice by reducing hepatic ferroptosis through regulation of the sirtuin 1 (SIRT1) signaling pathway [54]. Ferulic acid similarly protected against LPS‐induced hepatocyte injury in sheep through modulation of multiple ferroptosis‐associated markers, including ACSL4, LPCAT3, ALOX15, six‐transmembrane epithelial antigen of prostate 3 (STEAP3), GPX4, and glutamate‐cysteine ligase catalytic subunit (GCLC), thereby protecting hepatocytes from injury [55].

In another study, alpinetin reduced LPS/D‐GalN‐induced liver injury in mice by increasing the expression of ferroptosis‐inhibiting proteins, including NRF2, ferritin heavy chain (FTH), SLC7A11, and GPX4. The protective effects of alpinetin were shown to depend on the NRF2/SLC7A11/GPX4 signaling axis [56].

Phenolic compounds have additionally demonstrated protective effects against chemically induced liver injury caused by environmental toxins, pesticides, mycotoxins, and industrial chemicals. In fumonisin B1‐induced hepatotoxicity, apigenin ameliorated liver injury through the activation of the NRF2/FSP1 pathway [57]. Baicalin protected against aflatoxin B1‐induced liver injury by targeting the p53‐mediated ferroptosis pathway and modulating GPX4 and SLC7A11 expression [58].

Curcumin also attenuated ammonia‐induced liver injury in gibel carp by reducing ROS accumulation, lipid peroxidation, liver enzyme release, and ferroptosis‐associated gene dysregulation. Specifically, curcumin suppressed ferroptosis markers such as ACSL4 and prostaglandin‐endoperoxide synthase 2 (PTGS2) while restoring GPX4 expression [59]. Likewise, curcumin protected against polybrominated biphenyl (PBB)‐induced hepatotoxicity by restoring SLC7A11 expression and GSH synthesis through modulation of the KEAP1/NRF2/SLC7A11 axis [60].

Resveratrol protected hepatocytes against deoxynivalenol‐induced ferroptosis by restoring the redox balance, activating the SLC7A11/GSH/GPX4 pathway, and inhibiting ferroptosis [61].

Similarly, epicatechin alleviated emamectin benzoate‐induced liver injury through reduction of iron accumulation, restoration of GSH levels, and upregulation of GPX4, SLC7A11, NRF2, and HMOX1 [62].

Anthocyanins isolated from blueberries also demonstrated hepatoprotective effects against acrylamide‐induced toxicity in HepG2 cells. Anthocyanin treatment reduced oxidative stress and inhibited ferroptosis through the regulation of NRF2, GPX4, and SLC7A11, thereby alleviating liver injury [63].

Glyphosate exposure has also been linked to ferroptosis‐mediated hepatocyte injury. Grape seed‐derived procyanidin, a polymer composed of catechin and epicatechin monomers, protected against glyphosate‐induced ferroptosis through enhancement of NRF2/fibroblast growth factor 21 (FGF21) signaling crosstalk. Activation of this pathway enhanced antioxidant capacity, reduced oxidative stress, and suppressed ferroptotic injury [64].

Iron overload is another important contributor to ferroptosis‐associated liver injury. Administration of 1,2,3,4,6‐pentagalloyl glucose alleviated iron overload‐induced liver injury by reducing iron accumulation, oxidative stress, and ferroptosis in mice treated with iron dextran [65]. Similarly, ellagic acid attenuated iron overload‐induced ferroptosis in both mouse and cellular models. Transcriptomic analyses identified transforming growth factor beta (TGFβ)/Smad signaling as a major pathway underlying its protective effects [66].

Finally, licochalcone A, a flavonoid derived from *Glycyrrhiza uralensis*, protected RSL3‐treated HT1080 cells from ferroptosis. RNA sequencing and bioinformatic analyses demonstrated that licochalcone A suppressed ferroptosis through regulation of the specificity protein 1/DNA‐dependent protein kinase catalytic subunit axis, highlighting a potential link between DNA repair pathways and ferroptosis inhibition [67].

### 3.2. Triterpenoids

Triterpenoids are a diverse class of natural compounds composed of six isoprene units (30 carbon atoms; molecular formula: typically C_30_H_48_ for the parent triterpene) and have attracted increasing attention because of their antioxidant, anti‐inflammatory, and anti‐ferroptotic properties [68]. Emerging evidence suggests that several triterpenoids exert protective effects against ALF through the modulation of oxidative stress, iron homeostasis, and ferroptosis‐associated signaling pathways.

Ursolic acid, a pentacyclic triterpenoid, has demonstrated therapeutic potential in ALD by suppressing oxidative stress‐mediated ferroptosis and modulating the gut microbiota composition. Mechanistically, ursolic acid activated the liver kinase B1 (LKB1)/AMPK pathway, thereby inhibiting lipid synthesis and lipid peroxidation. In addition, ursolic acid upregulated key ferroptosis regulators, including GPX4 and SLC7A11, restoring antioxidant defenses and suppressing ferroptosis. Furthermore, ursolic acid improved alcohol metabolism and beneficially altered the gut microbiota disrupted by alcohol exposure [69].

In another experimental model, ursolic acid also protected Swiss albino mice against cisplatin‐induced liver damage. Treatment improved liver histological architecture, reduced oxidative stress, and inhibited ferroptosis by increasing TFR1 expression [70].

Similarly, ginsenoside Rd, a triterpenoid derived from ginseng, alleviated CCl_4_‐induced ALF in mice through the inhibition of ferroptosis. Ginsenoside Rd reduced levels of the lipid peroxidation marker 4‐HNE while restoring GSH and GPX4 levels. Importantly, administration of the ferroptosis inducer imidazole ketone erastin abolished these protective effects, confirming that ferroptosis suppression plays a central role in the hepatoprotective activity of ginsenoside Rd [71].

Consistent with these findings, ginsenoside‐Rg5, another ginseng‐derived triterpenoid, demonstrated protective effects against ALF in both in vivo and in vitro models. Ginsenoside‐Rg5 protected C57BL/6J mice and HepG2 cells from mitochondrial dysfunction and oxidative stress while simultaneously suppressing ferroptosis. Mechanistic investigations revealed that these effects were mediated through the modulation of the autophagy/NRF2 signaling pathway [72].

Oleanolic acid, a triterpenoid found in various fruits and medicinal plants, also protected against mercury chloride‐induced ALF by attenuating ferroptosis. Mercury exposure induced oxidative stress, iron overload, and dysregulation of the GPX4/SLC7A11 axis, whereas oleanolic acid restored antioxidant balance and iron homeostasis, thereby alleviating ferroptosis‐mediated hepatotoxicity [73].

Likewise, oral administration of saikosaponin D, a triterpenoid saponin, alleviated hepatic ischemia‐reperfusion injury in mice by suppressing ferroptosis through mitophagy. These findings further highlight the therapeutic potential of triterpenoids as modulators of ferroptosis in ALF [74].

### 3.3. Alkaloids

Alkaloids are a major class of nitrogen‐containing natural compounds that exhibit diverse pharmacological activities, including antioxidant, anti‐inflammatory, and anti‐ferroptotic effects. These compounds are typically alkaline because of the presence of nitrogen atoms, which are often incorporated into heterocyclic ring structures [75]. Increasing evidence suggests that several alkaloids exert hepatoprotective effects through the suppression of oxidative stress, mitochondrial dysfunction, and ferroptosis‐associated pathways.

Berbamine, a bisbenzylisoquinoline alkaloid extract, demonstrated protective effects against acetaminophen‐induced ALF. In both in vivo and in vitro models, berbamine treatment attenuated ferroptosis, mitochondrial dysfunction, and oxidative stress, thereby reducing liver injury [76].

Similarly, (+)‐clausenamide, an alkaloid derived from *Clausena lansium*, protected against acetaminophen‐induced ALF through the suppression of hepatocyte ferroptosis. Treatment with (+)‐clausenamide reduced lipid peroxidation, restored liver function, and modulated key ferroptosis‐associated markers, including downregulation of PTGS2 mRNA expression and upregulation of GPX4. Mechanistically, (+)‐clausenamide activated the NRF2 antioxidant pathway by directly modifying KEAP1 at cysteine residue 151, thereby preventing NRF2 degradation. This subsequently enhanced antioxidant defenses and suppressed ferroptosis [77].

### 3.4. Natural Products Derived From Traditional Chinese Medicine

Traditional Chinese medicine contains a wide range of bioactive compounds and herbal formulations with antioxidant, anti‐inflammatory, and cytoprotective properties. Increasing evidence suggests that natural products derived from traditional Chinese medicine can attenuate acute ALF through the modulation of oxidative stress, iron metabolism, and ferroptosis‐associated signaling pathways. This section summarizes several herbal extracts and natural compounds from traditional Chinese medicine that demonstrate hepatoprotective effects in experimental models of liver injury.

Extracts of *Rosa roxburghii* Tratt significantly ameliorated arsenic‐induced hepatotoxicity in rats through the regulation of the NRF2/GPX4 pathway. Arsenic exposure disrupted redox homeostasis, increased ROS generation, and promoted ferroptosis through the suppression of GPX4 activity. Treatment with the extract restored antioxidant defenses, normalized NRF2/GPX4 signaling, and inhibited ferroptosis, highlighting its potential as a natural therapeutic strategy for arsenic‐induced liver injury [78].

Similarly, Exocarpium Citri Grandis, a traditional Chinese medicinal extract, protected male C57BL/6J mice against LPS‐induced ALF. Exocarpium Citri Grandis administration reduced oxidative stress and suppressed ferroptosis through restoration of GPX4 and SLC7A11 expression levels [79].

Consistent with these findings, Jiedu Huayu Granules, a traditional Chinese herbal formulation, alleviated LPS‐induced ALF in mice. It reduced ferroptosis and enhanced the antioxidant capacity of hepatocytes, thereby ameliorating liver injury [80].

In another experimental model, Niujiaodihuang Detoxify Decoction (NDD) ameliorated hepatocyte injury induced by LPS/D‐GalN through suppression of ferroptosis. In liver cells, treatment with NDD restored mitochondrial function, reduced oxidative stress, increased the expression of SLC7A11, SLC3A2, and GPX4, and decreased ACSL4 expression, collectively contributing to protection against ferroptosis‐mediated ALF [81].

Protective effects against acetaminophen‐induced ALF have also been reported for *Artemisia keiskeana* Miq., a traditional Chinese medicinal herb. Its administration reduced pathological liver damage and increased NRF2 and GPX4 protein expression levels, indicating suppression of ferroptosis‐associated hepatotoxicity [82].

Likewise, Pien Tze Huang, a traditional Chinese medicinal formula, attenuated ALD in mice by suppressing oxidative stress and ferroptosis through modulation of the SIRT1/KEAP1/NRF2 signaling axis [83].

Gandankang, another traditional herbal extract, demonstrated protective effects against CCl_4_‐induced ALF in mice. Gandankang regulated key ferroptosis‐associated proteins, including GPX4 and ACSL4, while significantly reducing ROS production, Fe^2+^ accumulation, and lipid peroxidation. Mechanistically, these effects were mediated through the modulation of the KEAP1/NRF2 signaling pathway [84].

Similarly, Mori fructus aqueous extracts (MFAEs) exerted significant hepatoprotective effects against CCl_4_‐induced liver damage through the activation of the NRF2/HMOX1 pathway. MFAEs enhanced antioxidant defenses, including GSH and superoxide dismutase (SOD), while regulating ferroptosis‐related proteins such as ACSL4, SLC7A11, and GPX4. Importantly, inhibition of NRF2 abolished the protective effects of MFAEs, confirming the critical role of NRF2 signaling in their mechanism of action [85].

Further evidence demonstrated that *Euphorbia humifusa* Willd. ex Schltdl. (EHW) alleviated CCl_4_‐induced ALF in male mice through regulation of the KEAP1/NRF2 signaling axis. EHW significantly reduced serum ALT and AST levels, histological liver injury, iron accumulation, and lipid peroxidation. Network pharmacology analyses identified several bioactive compounds, including quercetin, kaempferol, and β‐sitosterol, that target ferroptosis‐ and oxidative stress‐related pathways. In addition, EHW restored iron homeostasis by decreasing TFR1 expression and increasing FTH1 levels while suppressing ACSL4 and ALOX12 expression [86].

Lastly, selenocystine, derived from *Cardamine hupingshanensis*, a perennial plant native to the Wuling Mountains of China, also protected against cadmium‐induced ALF in both HepG2 cells and mouse models. Selenocystine attenuated ferroptosis through modulation of the SLC7A11/GPX4 pathway, reduction of lipid peroxidation and ROS accumulation, and enhancement of detoxifying enzyme expression [87].

### 3.5. Miscellaneous Natural Products

In addition to phenolic compounds, triterpenoids, alkaloids, and traditional herbal formulations, several other plant‐derived compounds have demonstrated hepatoprotective effects through the modulation of ferroptosis‐associated pathways.

Lycopene, a carotenoid abundant in red fruits and vegetables, has demonstrated protective effects against multiple forms of toxin‐induced ALF through the suppression of ferroptosis. In atrazine‐induced hepatotoxicity, lycopene alleviated ferroptosis in hepatocytes by targeting cytochrome P450 oxidoreductase (CYPOR). Atrazine exposure increased CYPOR expression, resulting in lipid peroxidation and Fe^2+^ accumulation, whereas lycopene treatment suppressed CYPOR expression and reduced ferroptosis‐associated damage. Furthermore, CYPOR knockdown enhanced the protective effects of lycopene, identifying CYPOR as a potential regulator of ferroptosis in hepatocytes [88].

Consistent with these findings, lycopene also improved liver function and ferroptosis‐related markers in both in vitro and in vivo models of zearalenone‐induced hepatotoxicity. Mechanistic investigations demonstrated that these effects were mediated through the activation of the AMPK/NRF2 signaling pathway [89].

Similarly, Yang et al. demonstrated that ferroptosis contributes to hepatocyte toxicity induced by the T‐2 toxin. Lycopene treatment attenuated liver injury in mice through activation of the NRF2/mitophagy pathway, thereby suppressing ferroptosis and protecting hepatocytes from oxidative damage [90].

Spermidine, a naturally occurring polyamine, also protected mice against ethanol‐induced iron accumulation and ferroptosis. Spermidine regulated iron metabolism, suppressed ferritinophagy, and enhanced iron export from hepatocytes. Importantly, the protective effects of spermidine were dependent on NRF2 signaling as NRF2‐knockout mice failed to exhibit the beneficial effects of spermidine treatment [91].

α‐Lipoic acid, a potent free‐radical scavenger, effectively alleviated fluoride‐induced hepatocyte injury through inhibition of ferroptosis. Mechanistically, α‐lipoic acid modulated the SLC7A11/GPX4 signaling axis, reduced lipid peroxidation and iron accumulation, and restored the redox balance, ultimately attenuating hepatocyte injury in fluoride‐exposed mice [92].

Lastly, *Syzygium aromaticum* extract (SAE) protected against doxorubicin‐induced hepatotoxicity in male rats. Doxorubicin‐induced liver injury was strongly associated with ferroptosis, whereas SAE treatment restored biochemical liver function parameters and significantly reduced ferroptosis‐associated damage. Mechanistically, SAE activated the NRF2/SLC7A11/GPX4 signaling pathway, thereby enhancing antioxidant defenses and suppressing ferroptosis [93].

**Figure 1:**
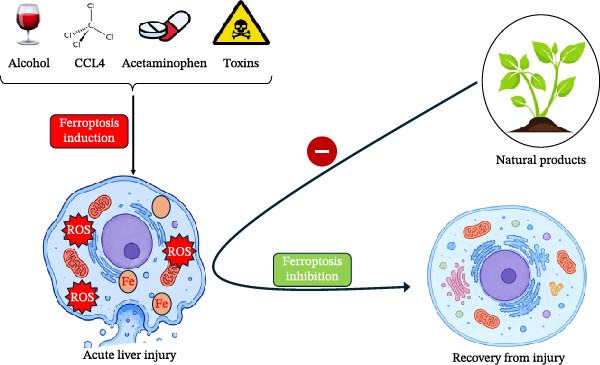
Schematic illustration of ferroptosis‐mediated acute liver injury and the protective role of natural products. Hepatotoxic insults such as alcohol, CCl_4_, acetaminophen, and other toxic agents promote ferroptosis through iron accumulation, ROS generation, and lipid peroxidation, leading to acute liver injury. Natural products can attenuate this process by inhibiting ferroptosis, thereby facilitating hepatocyte recovery and restoring cellular homeostasis. CCl_4_, carbon tetrachloride; ROS, reactive oxygen species.

Collectively, these studies demonstrate that diverse natural compounds, including phenolic compounds, triterpenoids, alkaloids, traditional Chinese herbal medicines, and other plant‐derived bioactive compounds, exert significant hepatoprotective effects across multiple models of ALF. Their protective activities are primarily mediated through suppression of ferroptosis, restoration of redox balance, regulation of iron metabolism, and modulation of antioxidant signaling pathways, particularly the NRF2/SLC7A11/GPX4 axis. Additional pathways, including AMPK, KEAP1, p53, TXNIP/NLRP3, TGFβ/Smad, and mitophagy‐related signaling, also contribute to their anti‐ferroptotic effects. Collectively, these findings highlight ferroptosis as an important therapeutic target and support the potential of natural products as promising therapeutic candidates for ALF (see Table 1 and Figure 1).

**Table 1:** Natural products modulating ferroptosis‐related pathways in ALF.

Inducer of ALF	Natural product	Molecular targets and pathways involved in ferroptosis modulation	References
Alcohol	Quercetin	↓ PERK‐dependent MAMs formation, ↑ GPX4, ↑SLC7A11, ↓PTGS2, ↓ ACSL4,	[41]
	Flavonoid glycosides (AH‐15)	↑ AMPK, ↑ NRF2, ↑ SOD, ↑ GSH, ↑SLC7A11	[42]
	Ursolic acid	↑ GPX4, ↑SLC7A11, ↑ LKB1/AMPK pathway	[69]
	Pien Tze Huang	↑ SIRT1/KEAP1/NRF2 axis, ↑ HMOX1	[83]
	Spermidine	↑ FTH1, ↓ ferritinophagy, ↑ NRF2	[91]
Acetaminophen	Catechin	↑ GPX4, ↑SLC7A11, ↓ ROS, ↓ MDA	[43]
	7,8‐dihydroxyflavone	↑ NRF2, ↑ GPX4, ↑ SLC7A11, ↓PTGS2, ↓ ACSL4	[44]
	Sesamin	↓ FOXO1, ↑ GPX4, ↑SLC7A11, ↓PTGS2	[45]
	Scutellarin	↑ GPX4, ↑SLC7A11, ↑ NRF2	[46]
	Sakuranetin	↓ ACSL4, ↑ GPX4, ↑ NRF2, ↑ SLC7A11	[47]
	Kaempferol	↑ GPX4, ↑ NRF2, ↓ KEAP1	[48]
	Rosmarinic acid	↑ Keap1/Nrf2/HMOX1, ↑ GPX4, ↑SLC7A11	[49]
	Berbamine	↑ NRF2/GCLC/GPX4 axis, ↑ FSP1	[76]
	(+)‐Clausenamide	↑ GPX4, ↑ NRF2, ↓ KEAP1, ↓PTGS2	[77]
	*Artemisia keiskeana* Miq.	↑ NRF2, ↑ GPX4, ↓ HMOX1, ↓ KEAP1, ↓ 5‐LOX	[82]
CCl4	Luteolin	↑ GPX4, ↑ GSH, ↓ ALOX12, ↑SLC7A11	[50]
	Schaftoside	↑ GPX4, ↑ NRF2, ↓ Mitochondrial damage	[51]
	Curcumin	↓ TXNIP, ↓ TFR1, ↑ GPX4, ↑SLC7A11, ↓TXNIP/NLRP3	[52]
	Flavonoids from astragali complanati Semen	↓ TFR1, ↓ VDAC3, ↓ Lipid peroxidation	[53]
	Ginsenoside Rd	↑ GPX4, ↑ GSH, ↓ Lipid peroxidation	[71]
	Gandankang	↑ GPX4, ↑ NRF2, ↓ KEAP1, ↓ ACSL4	[84]
	MFAEs	↑ HMOX1, ↑ GPX4, ↑ NRF2, ↓ ACSL4, ↑SLC7A11	[85]
	EHW	↓ TFR1, ↑ FTH1, ↓ ACSL4, ↓ ALOX12, ↓ PTGS2, ↑ NRF2	[86]
LPS/D‐GalN	Alpinetin	↓ TFR1, ↓ divalent metal transporter 1 (DMT1), ↑ FTH1, ↑ NRF2, ↑SLC7A11, ↑ GPX4	[56]
	Jiedu huayu granules	↑SLC7A11, ↑ GPX4, ↓ 5‐LOX‐catalyzed lipid peroxidation	[80]
	Niujiaodihuang detoxify decoction	↑ SLC7A11, ↑ GPX4, ↑ MFN1/2, ↓ ACSL4	[81]
LPS	Ferulic acid	↑ ACSL4, ↑ LPCAT3, ↑ ALOX15, ↑ STEAP3, ↑ GPX4, ↑ GCLC	[55]
	Ginsenoside Rg5	↓ ROS, ↑ SOD, ↑ GSH, ↑ GPX4, ↑ FTH1, ↑ FSP1, ↑ NRF2	[72]
	Exocarpium Citri Grandis	↑ NRF2, ↑ GPX4, ↑ SLC7A11	[79]
Sepsis	Isoferulic acid	↑ SIRT1, ↑ Nrf2, ↑SLC7A11, ↑ GPX4	[54]
Fumonisin B1	Apigenin	↑ Nrf2/FSP1 pathway, ↑ CoQ10H2	[57]
Aflatoxin B1	Baicalin	↓ P53, ↑SLC7A11, ↑ GPX4	[58]
Ammonia	Curcumin	↓ ACSL4, ↓ PTGS2, ↑ FPN, ↑ FTH1, ↑ SLC7A11, ↑ GPX4, ↑ NRF2	[59]
PBBs	Curcumin	↑ FTH1, ↑ SLC7A11, ↑ GPX4, ↑ GSH, ↑ NRF2, ↓ KEAP1	[60]
Deoxynivalenol	Resveratrol	↓ TFR1, ↑ GPX4, ↑ SLC7A11, ↑ GCLC, ↑ NRF2	[61]
Emamectin benzoate	Epicatechin	↑ HMOX1, ↑ GPX4, ↑ NRF2, ↑ SLC7A11	[62]
Acrylamide	Anthocyanins isolated from blueberries	↑ NRF2, ↓ KEAP1, ↑ HMOX1, ↑SLC7A11, ↑ GPX4, ↑ GSH, ↓ ACSL4	[63]
Glyphosate	Grape seed‐derived procyanidin	↑ NRF2, ↑ FGF21	[64]
Dextrose iron or RSL3	1,2,3,4,6‐Pentagalloyl glucose	↓ FTH1, ↑ TFR1, ↓ iron overload	[65]
Iron dextran or ferric ammonium citrate	Ellagic acid	↓ hepatic TGFβ, ↓ p‐Smad expression	[66]
RSL3	Licochalcone A	↑ transcription factor SP1, ↑ SP1/DNA‐PKcs axis, ↓ lipid peroxidation	[67]
Cisplatin	Ursolic acid	↓ ROS, ↑ SOD, ↑ GPX4, and ↑ GSH, ↑ TFR1	[70]
Mercury chloride	Oleanolic acid	↑ GPX4, ↑ SLC7A11, ↑ NRF2, ↑ SOD1, ↓ TFR1	[73]
Ischemia‐reperfusion injury	Saikosaponin D	↑ GSH, ↓ MDA, ↑SLC7A11, ↓ TFR1, ↑ GPX4, ↑ STAT3/PINK1/PARKIN	[74]
Arsenic	Extracts of *Rosa roxburghii* Tratt	*↑ NRF2*, ↑ GPX4	[78]
Cadmium	Selenocystine	↓ ACSL4, ↑ GPX4, ↑ SLC7A11	[87]
Atrazine	Lycopene	↓ CYPOR, ↓ Lipid peroxidation, ↓ Fe^2+^	[88]
Zearalenone	Lycopene	↑ NRF2, ↑ GPX4, ↑ FTH1, ↑ GSH	[89]
T‐2 toxin	Lycopene	↑ NRF2, ↑ GPX4, ↑ FTH1, ↑ PINK1, ↑ PARKIN	[90]
Fluoride	α‐lipoic acid	↑ GPX4, ↑ SLC7A11, ↓ Lipid peroxidation	[92]
Doxorubicin	SAE	↑ GPX4, ↑ NRF2, ↑SLC7A11	[93]

*Note:* Natural products modulating ferroptosis in acute liver failure (ALF) induced by various agents. This table summarizes natural compounds that regulate ferroptosis pathways across different ALF inducers, highlighting their molecular targets and mechanisms of action. Symbols: ↑, Upregulation/Increase; ↓, Downregulation/Decrease.

Abbreviations: 5‐LOX, 5‐lipoxygenase; ACSL4, acyl‐CoA long‐chain family member 4; AH‐15, flavonoid glycoside compound; ALF, acute liver failure; ALOX12, arachidonate 12‐lipoxygenase; ALOX15, arachidonate 15‐lipoxygenase; AMPK, AMP‐activated protein kinase; CoQ10H2, coenzyme Q10; CYPOR, cytochrome P450 oxidoreductase; DMT1, divalent metal transporter 1; DNA‐PKcs, DNA‐dependent protein kinase catalytic subunit; EHW, *Eclipta prostrata* water extract; Exocarpium Citri Grandis, *Citrus grandis* fruit peel; Fe^2+^, ferrous iron; FGF21, fibroblast growth factor 21; FOXO1, Forkhead box protein 1; FPN, ferroportin; FSP1, ferroptosis suppressor protein 1; FTH1, ferritin heavy chain 1; Gandankang, traditional Chinese medicine formula; GCLC, glutamate‐cysteine ligase catalytic subunit; GPX4, glutathione peroxidase 4; GSH, glutathione; HMOX1, heme oxygenase 1; Jiedu Huayu Granules, traditional Chinese medicine formula; KEAP1, Kelch‐like ECH‐associated protein 1; LPCAT3, lysophosphatidylcholine acyltransferase 3; MAMs, mitochondria‐associated membranes; MFAEs, *Moringa oleifera* flower aqueous extracts; MFN1/2, mitofusin 1/2; MDA, malondialdehyde; NLRP3, NOD‐like receptor family pyrin domain‐containing 3; NRF2, nuclear factor erythroid 2‐related factor 2; Niujiaodihuang Detoxify Decoction, traditional Chinese medicine formula; p‐Smad, phosphorylated Smad; PARKIN, Parkin RBR E3 ubiquitin‐protein ligase; PBBs, polyphenol‐rich berry extracts; PERK, protein kinase R‐like ER kinase; PINK1, PTEN‐induced kinase 1; PTGS2, prostaglandin‐endopeptidase 2; ROS, reactive oxygen species; SAE, Satureja extensa extract; SIRT1, Sirtuin 1; SLC7A11, solute carrier family 7 member 11; SOD, superoxide dismutase; SP1, transcription factor SP1; STAT3, signal transducer and activator of transcription 3; STEAP3, Steap3 endosomal metal transporter; TFR1, transferrin receptor 1; TGFβ, transforming growth factor beta; TXNIP, thioredoxin‐interacting protein; VDAC3, voltage‐dependent anion‐selective channel protein 3.

## 4. Chronic Liver Diseases

NAFLD is the most common chronic liver‐related disorder globally, characterized by the accumulation of excessive lipids in the liver, and represents a significant medical challenge. Current management methods primarily include insulin sensitizers, lipid‐lowering agents, and hepatoprotective agents, which help manage the symptoms rather than addressing the root causes. If left untreated, NAFLD can progress to more severe conditions such as NASH, liver fibrosis, and cirrhosis [94].

Recent studies have added a new dimension to understanding NAFLD by implicating ferroptosis in its pathogenesis. Luo et al. demonstrated that ferroptosis plays a critical role in NAFLD progression, where increased extracellular iron levels trigger ferroptosis by reducing GSH and GPX4, leading to lipid peroxidation and lipid droplet accumulation, both hallmarks of the disease. The study further showed that treatment with deferoxamine, a ferroptosis inhibitor, lowered iron levels, improved β‐oxidation, and enhanced lipid metabolism, thereby suggesting that targeting ferroptosis may represent a promising therapeutic strategy for NAFLD [95].

NASH is the severe form of NAFLD, characterized by excessive steatosis, chronic inflammation, and neutrophil infiltration. Currently, the main approach to treating NASH is weight loss through lifestyle changes as no specific medications are available. The pathogenesis of NASH involves complex mechanisms, including alterations in the gut microbiota, lipotoxicity from free fatty acids, mitochondrial dysfunction, and ER stress. NASH can progress to cirrhosis and has become a leading cause of liver transplants due to its rising prevalence and the lack of approved treatments [96]. Recently, ferroptosis has also been implicated in the pathogenesis of NASH. Shu et al. demonstrated that ferroptosis inhibition can alleviate the disease symptoms. In particular, suppressing ferroptosis reduces oxidative damage, lipid peroxidation, and liver injury, leading to improved liver function. These findings highlight ferroptosis as a key mechanism in NASH development and suggest that targeting ferroptosis could be a potential therapeutic strategy [97].

Liver fibrosis is a progressive chronic condition characterized by the excessive accumulation of extracellular matrix proteins, leading to the disruption of liver architecture and impaired function. In this condition, HSCs become activated and differentiate into myofibroblasts, which then produce collagen type I, contributing to liver scarring. Although the precise mechanisms behind liver fibrosis remain incompletely understood, several key factors drive its progression. These include the activation of HSCs, inflammation, oxidative stress, steatosis, aging, and the infiltration of immune cells, particularly macrophages. Liver fibrosis can also be triggered by various factors, such as chronic infections like hepatitis B and C, alcohol consumption, and rare conditions like hemochromatosis [98].

Currently, there are no specific antifibrotic drugs available, and advanced liver fibrosis remains largely incurable. Liver transplantation is the only effective treatment, but its high cost and the potential for postoperative immune rejection present significant challenges. These limitations emphasize the urgent need for innovative therapeutic strategies to manage liver fibrosis. If left untreated, liver fibrosis can progress to cirrhosis or even to HCC [99].

Emerging research has also linked ferroptosis to liver fibrosis as well. HSC activation leads to the deposition of the extracellular matrix, which exacerbates liver fibrosis progression. Inducing ferroptosis in HSCs has been shown to reduce liver fibrosis, making ferroptosis a promising therapeutic target for its treatment. Cao et al. [100] demonstrated that mesenchymal stem cell‐derived exosomal miR‐26a alleviates liver fibrosis by inducing ferroptosis and suppressing HSC activation.

HCC is a severe malignancy that arises from chronic liver diseases, often due to viral hepatitis, excessive alcohol intake, or metabolic disorders such as NAFLD. Its prevention, diagnosis, and treatment remain complex, and unfortunately, about half of HCC cases are still diagnosed at an advanced stage, with a recurrence rate of ~70% within 5 years of initial therapy [101].

Sorafenib has been widely used in advanced HCC due to its broad ability to suppress tumor proliferation and angiogenesis by targeting multiple signaling pathways. However, despite offering improved survival in some patients, its clinical benefit is limited to about 30%, with many developing resistance within 6 months and experiencing significant side effects. Alternative treatments such as lenvatinib, regorafenib, cabozantinib, and ramucirumab have been actively studied for second‐line use after sorafenib failure. Nonetheless, these agents also face issues of resistance, and their efficacy and safety remain major concerns [101, 102].

Given these limitations, there has been a growing interest in identifying new therapeutic strategies. In recent years, ferroptosis has emerged as a promising target in aggressive cancers like HCC. An expanding body of research underscores the pivotal role of ferroptosis in eliminating cancer cells and suppressing tumor progression. Iron overload, coupled with lipid peroxidation and ROS, all hallmarks of ferroptosis, compromise the integrity of tumor cell membranes, thereby impeding the invasion and metastasis of HCC cells and offering tangible clinical value in the management and prevention of HCC. Additionally, ferroptosis acts as a natural tumor‐suppressive mechanism, contributing to anticancer effects through the activation of various tumor suppressor pathways. Furthermore, studies have indicated that it can also enhance the sensitivity of HCC cells to treatments such as sorafenib, thereby improving therapeutic efficacy [103].

In conclusion, accumulating evidence from experimental models indicates that ferroptosis is a key pathogenic mechanism in the pathogenesis of chronic liver diseases and that its modulation may yield promising therapeutic outcomes for patients. In this section, we review current evidence on how natural products influence ferroptosis‐associated pathways in various stages of chronic liver diseases and discuss their therapeutic implications.

### 4.1. Phenolic Compounds

Phenolic compounds target ferroptosis across a wide spectrum of chronic liver diseases and have emerged as promising therapeutic candidates. Their beneficial effects are highly context‐dependent, reflecting the distinct roles of ferroptosis in different hepatic cell types and disease states. In metabolic liver diseases such as NAFLD and NASH, phenolic compounds primarily exert hepatoprotective effects by suppressing ferroptosis in hepatocytes. In contrast, during liver fibrosis and HCC, many phenolic compounds promote ferroptosis in activated HSCs or malignant hepatocytes, thereby limiting fibrogenesis and tumor progression.

In NAFLD, several phenolic compounds, including puerarin, oroxylin A, arbutin, hesperetin, silymarin, kaempferol, acacetin, epigallocatechin gallate, and resveratrol, have demonstrated protective effects through the modulation of ferroptosis‐related pathways. A recurring mechanism involves the activation of the NRF2/SLC7A11/GPX4 antioxidant axis, which plays a central role in maintaining cellular redox homeostasis and preventing ferroptotic cell death.

Puerarin, a well‐known isoflavone, ameliorated NAFLD by upregulating SIRT1, which subsequently enhanced NRF2 expression and nuclear translocation. Activation of this pathway mitigated ferroptosis in hepatocytes and improved glucose tolerance, insulin sensitivity, and liver function in experimental NAFLD models. Importantly, pharmacological inhibition of either SIRT1 or NRF2 diminished the protective effects of puerarin, confirming the critical involvement of this signaling cascade [104].

Similarly, oroxylin A inhibited ferroptosis through the activation of the KEAP1‐NRF2/GPX4/SLC7A11 pathway, thereby strengthening cellular antioxidant defenses. These effects were abolished in NRF2‐deficient mice, confirming the NRF2‐dependents activity. Furthermore, molecular docking and simulation analyses revealed that oroxylin A directly binds to KEAP1, promoting NRF2 stabilization and activation [105].

Hesperetin likewise alleviated HFD‐induced NAFLD through direct interaction with the Farnesoid X receptor (FXR), leading to increased expression of GPX4 and SLC7A11. In addition, hesperetin enhanced hepatic antioxidant capacity through upregulation of GCLC, thereby reinforcing GSH biosynthesis [106].

Silymarin also protected against lipotoxic injury in HFD‐fed rodents and palmitic acid‐treated HepG2 cells by restoring SLC7A11 expression, promoting cystine uptake, replenishing GSH levels, and ultimately suppressing ferroptosis [107].

Beyond direct regulation of antioxidant pathways, several phenolic compounds modulate ferroptosis through alternative mechanisms. Arbutin alleviated fatty liver pathology by targeting fat mass and obesity‐associated protein (FTO), which regulates the methylation status of SLC7A11, highlighting the therapeutic relevance of the FTO/SLC7A11 axis in NAFLD [108].

Kaempferol intervention successfully restored cell viability, suppressed pro‐inflammatory cytokines (tumor necrosis factor alpha [TNF‐α], interleukin [IL]‐6, IL‐1 beta [IL‐1β]), and neutralized ROS to halt the progression of NAFLD. Mechanistically, kaempferol achieved its hepatoprotective effects by activating the AMPK signaling pathway, thereby suppressing the downstream ferroptosis cascade. By simultaneously lowering lipid influx and halting lipid peroxidation, kaempferol hindered the pathological cycle of lipid overload and oxidative cell death [109].

Acacetin attenuated hepatic lipid accumulation, inhibited ER stress, suppressed ferroptosis, and downregulated lipogenic gene expression, indicating that ER stress functions as an upstream regulator of ferroptosis in NAFLD [110].

Mitochondrial dysfunction and hypoxia‐related signaling have also emerged as important contributors to ferroptosis during NAFLD progression. Epigallocatechin gallate protected mice against HFD‐induced NAFLD by targeting mitochondrial ROS‐mediated ferroptosis, reducing hepatic injury, lipid accumulation, and oxidative stress, thereby highlighting its potential as a mitochondria‐targeted anti‐ferroptotic agent [111].

Likewise, resveratrol significantly reduced inflammatory responses, attenuated hepatic tissue damage, and restored the mitochondrial ultrastructure by suppressing ferroptosis. Mechanistically, high‐altitude hypoxia aggravated NAFLD by stabilizing hypoxia‐inducible factor 1‐alpha (HIF‐1α), which subsequently upregulated ACSL4 and TFR1, promoting intracellular iron accumulation, lipid peroxide generation, and 4‐HNE production. Resveratrol interrupted this HIF‐1α/ACSL4/TFR1 signaling axis, thereby suppressing ferroptosis and providing a potential therapeutic strategy for metabolic liver disorders associated with hypoxic conditions [112].

A similar anti‐ferroptotic pattern has been observed in NASH. Several naturally occurring compounds, including icariin, tectorigenin, and hydroxytyrosol, have demonstrated therapeutic efficacy through the suppression of ferroptosis. In methionine‐choline‐deficient (MCD) diet‐induced NASH, icariin significantly reduced serum ALT and AST levels, ameliorated hepatic steatosis and inflammation, and suppressed ferroptosis. These effects were accompanied by reduced hepatic iron, MDA, and 4‐HNE levels, downregulated levels of ACSL4 and ALOX12, and activation of the NRF2/SLC7A11/GPX4 pathway, ultimately restored mitochondrial integrity [113].

Tectorigenin, a bioactive compound derived from *Iris tectorum*, also inhibited ferroptosis in NASH. Mechanistically, its protective effects were mediated by upregulation of the tRNA‐derived fragment tRF‐31R9J, which suppressed pro‐ferroptotic genes including activating transcription factor 3 (ATF3), ATF4, and cation transport regulator like 1 (CHAC1), thereby reducing ferroptosis and enhancing cell viability [114].

Similarly, hydroxytyrosol, a major polyphenol found in extra virgin olive oil, alleviated hepatic steatosis and inflammation in long‐term HFD‐induced NASH and palmitic acid‐treated hepatocytes. It exerted its anti‐ferroptotic effects by preventing suppressor of cytokine signaling 2 (SOCS2)‐mediated ubiquitination and degradation of SLC7A11, thereby restoring SLC7A11 and GPX4 protein levels and suppressing ferroptosis [115].

In contrast to NAFLD and NASH, where ferroptosis suppression is beneficial, liver fibrosis is largely driven by activated HSCs. Consequently, many phenolic compounds exert antifibrotic effects through selective induction of ferroptosis in these cells, thereby reducing extracellular matrix deposition and fibrogenesis.

Several compounds promote HSC ferroptosis by disrupting iron homeostasis. Ellagic acid induced ferroptosis by impairing soluble N‐ethylmaleimide‐sensitive factor attachment protein receptor (SNARE) complex formation involving VAMP2/syntaxin 4 and vesicle‐associated membrane protein 2 (VAMP2)/synaptosome‐associated protein 23 (SNAP23), which reduced FPN translocation and iron export. The resulting intracellular iron accumulation triggered ferroptosis, and modulation of VAMP2 expression significantly influenced its antifibrotic efficacy [116].

Astragalin similarly promoted ferroptosis through the induction of ferritinophagy, a selective autophagic process mediated by nuclear receptor coactivator 4 (NCOA4) that degrades ferritin. This process generated intracellular iron overload, increased lipid peroxidation, and promoted HSC death. Consistent with this mechanism, astragalin increased the expression of PTGS2, TFR1, and ACSL4 while suppressing GPX4 expression [117].

Other phenolic compounds primarily target the GPX4/SLC7A11 ferroptosis defense system. Baicalein attenuated CCl4‐induced liver fibrosis by downregulating GPX4 through scavenger receptor class A member 5 (SCARA5)‐mediated signaling, thereby promoting ferroptosis in HSCs [118].

Danshensu similarly induced ferroptosis in HSCs by increasing lipid ROS levels and modulating GPX4 and SLC7A11 expression. The antifibrotic effects of danshensu were abolished by liproxstatin‐1, confirming the central role of ferroptosis in its activity [119].

Wogonoside, a flavonoid from Scutellaria baicalensis, selectively induced ferroptosis through the SOCS1/P53/SLC7A11 pathway, reducing α‐smooth muscle actin (α‐SMA) expression, depleting SLC7A11, GPX4, and GSH, and increasing iron, ROS, and MDA levels. These effects were reversed by ferroptosis inhibitors or blockade of SOCS1 or P53 signaling [120].

Additional compounds induce ferroptosis mainly through metabolic reprogramming and redox regulation. Treatment with *Ficus hirta* Vahl, a natural product from the Moraceae family, reduced fibrosis and inflammation by modulating the GSH metabolism, a critical regulator of ferroptosis. Apigenin was identified as a major active constituent responsible for these effects [121].

Formononetin promoted ferroptosis in activated HSCs through direct targeting of NADPH oxidase 4 (NOX4), increasing NADPH oxidase activity, elevating the NADP+/NADPH ratio, and triggering ferroptotic cell death. Inhibitors of ferroptosis abolished its effects, confirming the mechanism of action [122].

Resveratrol also induced ferroptosis in activated HSCs by suppressing ATF4‐mediated ER stress signaling and blocking glutamine uptake required for GSH synthesis. The resulting depletion of GPX4 and accumulation of iron promoted ferroptotic cell death and attenuated fibrosis [123].

Similarly, rubimaillin induced ferroptosis through direct inhibition of carnitine palmitoyltransferase 1A (CPT1A) by binding to SER592, THR594, and THR689 residues. This interaction triggered profound metabolic reprogramming in activated HSCs, ultimately leading to ferroptosis. Importantly, CPT1A overexpression completely abolished these effects, confirming the central role of the rubimaillin‐CPT1A axis in its antifibrotic activity [124].

The induction of ferroptosis also represents a promising therapeutic strategy in HCC, where malignant hepatocytes often develop resistance to apoptosis. Several phenolic compounds have demonstrated antitumor activity through selective activation of ferroptotic pathways.

A major therapeutic target in HCC is the NRF2/SLC7A11/GPX4 antioxidant system. Licochalcone A suppressed HCC growth by downregulating SLC7A11, thereby inhibiting the GSH‐GPX4 antioxidant defense pathway, promoting ROS accumulation, and triggering ferroptosis [125].

Similarly, sappanone A induced ferroptosis by suppressing NRF2, SLC7A11, and GPX4 expression. In addition, it directly targeted inosine monophosphate dehydrogenase 2 (IMPDH2), revealing a novel regulatory mechanism and potential therapeutic target in HCC [126].

Curcumin likewise induced ferroptosis in both xenograft models and human HCC cell lines by suppressing P62 and NRF2 while increasing KEAP1 expression. This inhibition of the P62–KEAP1–NRF2 signaling axis promoted lethal lipid peroxidation and ferroptotic cell death, effects that could be reversed by ferrostatin‐1 or genetic activation of P62 [127].

Additional mechanisms have also been identified. Emodin inhibited tumor growth, depleted GSH, disrupted mitochondrial membrane potential, and induced lipid peroxidation through the upregulation of miR‐4465. This microRNA directly suppressed NRF3, resulting in the inactivation of the NRF3/3‐hydroxy‐3‐methylglutaryl‐CoA reductase (HMGCR)/GPX4 signaling pathway and induction of ferroptosis [128].

Macelignan exerted antitumor effects by remodeling the tumor immune microenvironment. Specifically, it suppressed SLC7A11, depleted GSH, reduced GPX4 expression, and induced ferroptosis in tumor‐associated macrophages. This disrupted the signal transducer and activator of transcription 6 (STAT6)/peroxisome proliferator‐activated receptor gamma (PPARγ)/Krüppel‐like factor 4 (KLF4) signaling cascade and reduced production of the pro‐tumor chemokine C–C motif chemokine ligand 2 (CCL2), ultimately suppressing tumor growth and epithelial–mesenchymal transition without detectable systemic toxicity [129].

Collectively, these findings demonstrate that phenolic compounds exert disease‐ and cell‐specific regulation of ferroptosis across chronic liver diseases.

### 4.2. Terpenoids

Terpenoids have demonstrated therapeutic potential across a diverse range of chronic liver diseases, including NAFLD, NASH, liver fibrosis, and HCC, largely through the modulation of ferroptosis‐related pathways. Similar to phenolic compounds, terpenoids exhibit context‐dependent regulation of ferroptosis, suppressing ferroptotic cell death in metabolic liver disorders while promoting ferroptosis in activated HSCs and malignant liver cells to attenuate fibrosis and tumor progression.

In NAFLD, multiple terpenoids, including ginkgolide B, diosgenin, dimeric guaianolide sesquiterpenoids, Hypercohin A, and Paris Saponin VII, have demonstrated hepatoprotective effects through suppression of ferroptosis. A common mechanism underlying these effects involves the activation of antioxidant signaling pathways, particularly the NRF2 axis. Ginkgolide B reduced hepatic lipid accumulation and alleviated liver injury, oxidative stress, and iron overload through activation of NRF2 signaling, which subsequently enhanced the expression of ferroptosis‐regulating proteins, including GPX4 and HMOX1 [130].

Similarly, diosgenin, a naturally occurring steroidal saponin, attenuated NAFLD by activating NRF2 signaling, resulting in reduced lipid accumulation and oxidative stress while increasing the expression of antioxidant enzymes [131].

Additional terpenoids exert anti‐ferroptotic effects through the modulation of metabolic regulators. Dimeric guaianolide sesquiterpenoids, particularly the novel compound chryindicolide O, reduced hepatic steatosis in both lipid‐overloaded hepatocytes and HFD‐fed zebrafish by directly binding to and activating the deacetylase SIRT1. Activation of SIRT1 suppressed de novo lipogenesis, enhanced fatty acid β‐oxidation, and concurrently inhibited ferroptosis, thereby improving hepatic lipid metabolism [132].

Likewise, Paris Saponin VII, a bioactive constituent isolated from the rhizomes of *Paris polyphylla*, protected HFD‐induced rats and oleic acid‐treated HepG2 cells from ferroptosis by reducing ferroptosis‐associated markers, including Fe^2+^, MDA, ROS, TFR1, and ACSL4, while simultaneously upregulating SLC7A11 and GPX4 expression. Mechanistically, its protective effects were mediated through interaction with ubiquitin‐specific peptidase 7 (USP7) as pharmacological inhibition of USP7 abolished its beneficial actions [133].

A similar anti‐ferroptotic pattern occurs in NASH models. Hinokitiol, a bioactive monoterpene, effectively alleviated NASH through the activation of the NRF2/GPX4 signaling pathway. Hinokitiol supplementation increased NRF2 and GPX4 expression, improved lipid metabolism, reduced lipid peroxidation, and suppressed ferroptosis‐related markers. Importantly, genetic silencing of NRF2 reversed these protective effects, confirming the central role of NRF2 signaling in mediating the anti‐ferroptotic activity of hinokitiol [134].

In contrast to metabolic liver diseases, where ferroptosis suppression is beneficial, induction of ferroptosis in activated HSCs has emerged as an effective strategy for limiting liver fibrosis. Several naturally occurring terpenoids have been shown to exert antifibrotic effects through selective promotion of ferroptosis in these cells.

Ginsenoside Rg3, one of the major bioactive constituents of *Panax ginseng*, attenuated liver fibrosis by promoting ferroptosis in HSCs. Ginsenoside Rg3 treatment induced intracellular iron accumulation, GSH depletion, and lipid peroxidation. Furthermore, it restored the expression of ACSL4, a key mediator of ferroptosis, through demethylation of the ACSL4 gene, highlighting an epigenetic mechanism underlying its antifibrotic activity [135].

Similarly, dihydrotanshinone, a diterpenoid quinone, promoted ferroptosis through the upregulation of ALOX15 in HSCs. This effect was mediated by the increased expression of early growth response 1 (EGR1), which suppressed DNA methyltransferase 1 (DNMT1), a negative regulator of ALOX15. The resulting demethylation and activation of ALOX15 enhanced ferroptosis through the EGR1/DNMT1/ALOX15 signaling axis [136].

Several terpenoids also target the SLC7A11‐dependent ferroptosis defense system. Glycyrrhetic acid 3‐O‐mono‐β‐d‐glucuronide (GAMG) alleviated liver fibrosis by targeting inflammatory macrophages through the interferon regulatory factor 1 (IRF1)/SLC7A11 pathway. GAMG treatment reduced hepatocyte steatosis and inflammatory infiltration while selectively modulating macrophage populations through upregulation of IRF1 and suppression of the ferroptosis inhibitor SLC7A11 [137].

Likewise, Ginsenoside Rh2 induced ferroptosis in activated HSCs through upregulation of IRF1, which directly binds to the SLC7A11 promoter and suppresses its expression. This mechanism promoted ferroptosis, inhibited HSC activation, reduced macrophage recruitment, and attenuated hepatic inflammation [138].

Other terpenoids induce ferroptosis through the modulation of oxidative stress and autophagy‐related pathways. Celastrol promoted ferroptosis in activated HSCs by increasing ROS generation and lipid peroxidation through the inhibition of antioxidant peroxiredoxins (PRDX1, PRDX2, PRDX4, and PRDX6) while simultaneously inducing HMOX1 expression. Knockdown studies confirmed the essential contribution of these targets to celastrol‐mediated ferroptosis and antifibrotic activity [139].

Similarly, Ginsenoside Rb1 (GRb1) induced ferroptosis in HSCs, resulting in reduced α‐SMA expression, increased GSH depletion, elevated MDA production, iron overload, and ROS accumulation. Mechanistically, GRb1 upregulated Beclin 1 while suppressing the SLC7A11 expression. Silencing Beclin 1 reversed ferroptosis induction and restored SLC7A11 expression, demonstrating the importance of the Beclin 1/SLC7A11 signaling axis in GRb1‐mediated antifibrotic effects [140].

The induction of ferroptosis has also emerged as a promising therapeutic strategy for HCC, where malignant hepatocytes frequently develop resistance to apoptosis. Numerous terpenoids have demonstrated antitumor activity through the disruption of ferroptosis defense systems and enhancement of iron‐dependent oxidative damage.

Several terpenoids induce ferroptosis through the suppression of the SLC7A11/GPX4 antioxidant network. Saikosaponin A, a natural triterpenoid saponin, triggered ER stress, leading to ATF3 upregulation and subsequent repression of the SLC7A11 expression. This cascade increased intracellular iron and MDA levels, depleted GSH, enhanced lipid peroxidation, and ultimately induced ferroptosis, thereby suppressing HCC progression through the ER stress/ATF3/SLC7A11 signaling pathway [141].

Similarly, dihydroartemisinin promoted ferroptosis by inhibiting the ATF4/SLC7A11 pathway. Since ATF4 directly binds to the SLC7A11 promoter and contributes to ferroptosis resistance, suppression of ATF4 reduced SLC7A11 expression, increased lipid peroxidation, and induced ferroptotic cell death in HCC cells [142].

Other terpenoids target upstream regulators of antioxidant defense pathways. SSPH I, a steroidal saponin, induced ferroptosis through activation of extracellular signal‐regulated kinase (ERK) 1/2 signaling, which subsequently downregulated the NRF2/HMOX1 axis. Activation of this pathway promoted iron accumulation and ROS production, resulting in mitochondrial dysfunction and reduced viability of HCC cells [143].

Oridonin likewise induced ferroptosis through the direct targeting of HMOX1. Specifically, oridonin enhanced HMOX1 stability by blocking its ubiquitin‐mediated degradation at the lysine residue K86. Sustained HMOX1 activity promoted intracellular ferrous iron accumulation, oxidative stress, and ferroptotic cell death in malignant hepatocytes [144].

A number of terpenoids directly regulate the NRF2/HMOX1/GPX4 signaling network. Polyphyllin I inhibited HCC progression by inducing mitochondrial dysfunction and ferroptosis through direct interaction with NRF2, HMOX1, and GPX4. This led to iron accumulation, GSH depletion, elevated ROS production, mitochondrial structural damage, and reduced mitochondrial membrane potential [145].

Similarly, monotropein induced ferroptosis through suppression of the NRF2/HMOX1/GPX4 pathway, resulting in enhanced lipid peroxidation and intracellular iron accumulation [146].

Polyphyllin VI also promoted ferroptosis by suppressing the STAT3/GPX4 signaling axis. Inhibition of STAT3 phosphorylation and nuclear translocation reduced GPX4 expression, increased lipid peroxidation, and ultimately inhibited HCC cell proliferation, invasion, and metastasis [147].

Ginsenoside compound K (CK), a major metabolite of ginseng saponins, has been reported to induce ferroptosis through multiple complementary mechanisms. In one study, CK was shown to promote the degradation of GPX4, leading to its reduced expression. Loss of GPX4 triggered extensive lipid peroxidation and ferroptotic cell death [148].

In a separate study, CK facilitated ferroptosis through the modulation of the FOXO pathway. Specifically, CK inhibited FOXO1 phosphorylation, resulting in decreased expression of GPX4 and SLC7A11. Consistent with these molecular changes, CK significantly suppressed tumor growth in HepG2 xenograft models [149].

Parthenolide, a naturally occurring sesquiterpene lactone, represents another potent ferroptosis inducer in HCC. It promoted rapid thiol oxidation, accelerated GSH depletion, reduced GPX4 protein levels, and increased lipid peroxidation and mitochondrial dysfunction. Through these mechanisms, parthenolide sensitized HCC cells to ferroptosis and significantly reduced tumor cell viability and survival [150].

Collectively, these findings demonstrate that terpenoids exert substantial hepatoprotective and antitumor activities through context‐dependent modulation of ferroptosis. In NAFLD and NASH, terpenoids predominantly suppress ferroptosis, and in liver fibrosis and HCC, they promote ferroptosis in activated HSCs and malignant hepatocytes.

### 4.3. Alkaloids

Alkaloids have also emerged as promising modulators of ferroptosis in chronic liver diseases, particularly liver fibrosis and HCC. Several alkaloids exert therapeutic effects by promoting ferroptosis in activated HSCs or malignant hepatocytes, thereby limiting fibrosis progression and tumor growth.

In liver fibrosis, magnoflorine and berberine have demonstrated significant antifibrotic activity through the induction of ferroptosis in HSCs. Magnoflorine, a naturally occurring alkaloid, attenuated liver fibrosis by promoting ferroptosis in activated HSCs, resulting in reduced liver injury, collagen deposition, and HSC activation. Mechanistically, magnoflorine suppressed the TGF‐β/Smad signaling pathway, a central regulator of fibrogenesis. Notably, magnoflorine preferentially induced ferroptosis in ROS‐activated HSCs, highlighting its selective therapeutic potential in fibrotic liver disease [151].

Similarly, berberine alleviated liver fibrosis in experimental models through ferroptosis induction in HSCs. Berberine increased intracellular ROS production, which promoted ferritin degradation and subsequent ferrous iron accumulation. The resulting iron overload enhanced lipid peroxidation and triggered ferroptotic cell death, ultimately reducing fibrogenesis. Importantly, treatment with the ferroptosis inhibitor ferrostatin‐1 significantly attenuated the antifibrotic effects of berberine, confirming that its therapeutic activity is largely dependent on ferroptosis induction [152].

In HCC, solasonine has emerged as a potent ferroptosis‐inducing alkaloid with significant antitumor activity. Solasonine promoted ferroptosis by disrupting the GSH‐dependent antioxidant defense system through the suppression of GPX4 and GSH synthetase (GSS), two critical enzymes involved in detoxifying lipid peroxides and maintaining redox homeostasis. Inhibition of these protective mechanisms resulted in excessive lipid peroxidation and ferroptotic cell death. Consistent with these molecular effects, solasonine significantly inhibited tumor growth and metastasis, highlighting its therapeutic potential as a GPX4‐targeting ferroptosis inducer in HCC [153].

Collectively, these findings demonstrate that alkaloids exert beneficial effects in liver fibrosis and HCC primarily through the induction of ferroptosis.

### 4.4. Coumarins

Coumarins have emerged as promising ferroptosis‐inducing agents in chronic liver diseases, particularly liver fibrosis and HCC. Current evidence suggests that their therapeutic effects are predominantly mediated through the disruption of cellular antioxidant defenses, promotion of iron accumulation, and enhancement of lipid peroxidation, ultimately leading to ferroptotic cell death in activated HSCs or malignant hepatocytes.

In liver fibrosis, daphnetin demonstrated significant antifibrotic activity by selectively targeting activated HSCs. In a CCl_4_‐induced liver fibrosis model, daphnetin promoted ubiquitin‐mediated degradation of GPX4 while simultaneously stimulating ferritinophagy‐mediated ferroptosis. The combined loss of antioxidant protection and increased intracellular iron availability triggered extensive lipid peroxidation and iron overload within activated HSCs. Consequently, daphnetin significantly reduced collagen deposition and suppressed the expression of profibrotic genes, thereby attenuating liver fibrosis [154].

Several coumarins have also demonstrated potent antitumor effects in HCC through the induction of ferroptosis. Auraptene promoted ferroptotic cell death by facilitating the ubiquitin‐proteasomal degradation of SLC7A11, a critical component of the cystine/glutamate antiporter system responsible for maintaining intracellular GSH levels. Loss of SLC7A11 function resulted in increased total and lipid ROS accumulation, ultimately triggering ferroptosis [155].

Similarly, esculetin induced ferroptosis through the disruption of the NRF2/GPX4 signaling axis. Inhibition of this major antioxidant pathway increased intracellular Fe^2+^ accumulation and enhanced ROS generation through the Fenton reaction. The resulting oxidative stress promoted extensive lipid peroxidation, as demonstrated by elevated MDA levels and reduced GPX4 activity, ultimately suppressing HCC progression [156].

Decursin likewise exerted antitumor effects by inducing ferroptosis and impairing tumor cell motility. Mechanistically, decursin suppressed the NRF2/GPX4 antioxidant pathway, depleted intracellular GSH reserves, and promoted lipid peroxidation, thereby generating lethal oxidative stress within HCC cells. In addition, decursin interfered with iron homeostasis through modulation of the SLC11A2 pathway, further enhancing ferroptotic cell death and restraining tumor progression [157].

Collectively, these findings demonstrate that coumarins exert therapeutic effects in liver fibrosis and HCC primarily through the induction of ferroptosis.

### 4.5. Traditional Chinese Medicine

Traditional Chinese medicine has long been recognized for its ability to modulate multiple pathological pathways simultaneously. Increasing evidence suggests that the therapeutic effects of these bioactive compounds in chronic liver diseases are mediated, at least in part, through the regulation of ferroptosis. Depending on the disease context, these interventions either suppress ferroptosis to protect hepatocytes from injury or promote ferroptosis in pathological cell populations to limit the disease progression.

In metabolic liver diseases such as NAFLD and NASH, traditional Chinese formulations primarily exert hepatoprotective effects through suppression of ferroptosis and restoration of redox homeostasis. Exocarpium citri grandis formula granules (ECGFG), a citrus peel‐derived formulation, significantly improved hepatic steatosis in zebrafish models of NAFLD. ECGFG reduced hepatic lipid accumulation and oxidative stress while restoring iron homeostasis through regulation of TFR1, FPN, and key ferroptosis‐associated genes, including SLC7A11 and GPX4 [158].

Similarly, Xiaoyao San, a traditional Chinese herbal formulation, ameliorated NAFLD in chronic restraint‐stress‐induced animal models. Treatment reduced hepatic lipid accumulation and attenuated systemic inflammatory and oxidative stress responses. Mechanistically, Xiaoyao San upregulated PPARα and GPX4 while suppressing HMOX1 expression, thereby limiting hepatic iron accumulation and ferroptosis [159].

Likewise, Hu Gan Tang (HGT), a modified traditional Chinese medicinal formula, protected against NAFLD by suppressing iron‐dependent cell death and hepatic lipid accumulation. HGT reduced MDA and ROS levels, restored SOD activity, and promoted nuclear translocation of NRF2. Activated NRF2 subsequently enhanced the expression of key downstream antioxidant and ferroptosis‐regulating molecules, including SLC7A11, GPX4, and FTH1, thereby preventing hepatic ferroptosis [160].

The anti‐ferroptotic effects of the traditional Chinese formula have also been demonstrated in NASH. Shugan Xiaozhi (SGXZ) decoction improved iron metabolism, reduced lipid peroxidation, and enhanced SLC7A11 activity. Mechanistically, SGXZ acted through the p53/SLC7A11/GPX4 signaling pathway, a central regulatory axis of ferroptosis, resulting in marked suppression of ferroptotic cell death and attenuation of NASH progression [161].

Multiple traditional Chinese formulations have demonstrated efficacy against CCl_4_‐induced fibrosis through the modulation of oxidative stress and ferroptosis‐associated signaling pathways.

Paeoniae Radix Alba Polysaccharide attenuated liver fibrosis by reducing extracellular matrix deposition, inflammation, and tissue degeneration. Mechanistically, it simultaneously inhibited the phosphoinositide 3‐kinase (PI3k)/Protein Kinase B (PKB)/mammalian target of rapamycin (mTOR) signaling pathway while activating the SLC7A11/GPX4 antioxidant system. This coordinated regulation effectively suppressed ferroptosis and oxidative stress, thereby protecting the hepatic tissue from fibrotic injury [162].

Albiflorin, a monoterpene glycoside isolated from traditional Chinese *Paeonia lactiflora* Pall., significantly improved liver function, reduced serum ALT and AST levels, restored hepatic architecture, and suppressed fibrotic markers including α‐SMA, Collagen I, and Fibronectin. Mechanistic investigations demonstrated that albiflorin directly binds GPX4, forming a stable protein complex that activates the SLC7A11/GPX4 signaling pathway. This activation increased the expression of SLC7A11, GPX4, FTH1, and FTH1, while reducing TFR1 and HMOX1 expression, ultimately limiting ferroptosis and fibrotic progression [163].

Longchai Decoction, an empirical formulation, reduced liver fibrosis by suppressing ferroptosis and systemic inflammation. It decreased the circulating levels of IL‐1β, IL‐6, and TNF‐α while preserving mitochondrial structural integrity. Furthermore, it enhanced the expression of NRF2, GPX4, and HMOX1, thereby reducing iron accumulation and lipid peroxidation within the hepatic tissue [164].

Similarly, Yindanpinggan Capsule alleviated CCl_4_‐induced liver fibrosis by modulating ferroptosis‐associated pathways. Treatment improved liver function, reduced fibrosis indices, and restored tissue homeostasis. Mechanistically, it simultaneously regulated the PPARγ/GPX4 signaling pathway and the SLC7A11/GSH metabolic axis, resulting in reduced MDA accumulation, attenuation of ferrous iron overload, enhancement of GSH activity, and suppression of ferroptotic cell death. By restoring antioxidant defenses and reducing oxidative damage, the capsule effectively attenuated fibrotic progression [165].

Taohong Siwu Decoction also demonstrated antifibrotic activity in both CCl_4_‐induced murine fibrosis and erastin‐treated BRL‐3A cells. Treatment reduced fibrosis‐associated markers, decreased oxidative stress, regulated serum iron levels, and modulated the expression of key ferroptosis‐related molecules, including SLC7A11, NRF2, KEAP1, and GPX4. Collectively, these effects suppressed ferroptosis and attenuated liver fibrosis [166].

The regulation of ferroptosis by traditional Chinese formulas has also shown promise in HCC. Oxymatrine, a major bioactive component derived from traditional Chinese medicinal herbs, suppressed HCC progression through modulation of the SIRT1/Yin Yang 1 (YY1)/GPX4 signaling axis. Oxymatrine reduced cellular proliferation and promoted tumor cell death by suppressing the zinc‐finger transcription factor YY1 and GPX4, both of which contribute to ferroptosis resistance, while simultaneously increasing SIRT1 expression [167].

Similarly, Hua Zheng San Ji Fang (HZSJF), a traditional Chinese medicinal formula, inhibited HCC progression by promoting oxidative stress and ferroptosis. It increased KEAP1 expression, resulting in the suppression of the NRF2/HMOX1 antioxidant pathway and consequent loss of cellular antioxidant protection. This disruption promoted intracellular Fe^2+^ accumulation, enhanced lipid peroxidation, and depleted GSH and SOD levels, ultimately driving ferroptotic cell death in HCC cells [168].

Collectively, these studies demonstrate that traditional Chinese formulas exert therapeutic effects across multiple chronic liver diseases through the regulation of ferroptosis.

### 4.6. Miscellaneous Natural Products

Several natural products that do not fall within the major categories of phenolics, terpenoids, alkaloids, or coumarins have also demonstrated significant therapeutic potential in chronic liver diseases through the modulation of ferroptosis. Similar to other classes of natural compounds, these agents exert context‐dependent effects, predominantly suppressing ferroptosis in metabolic liver diseases while promoting ferroptosis in malignant cells.

In NAFLD, several miscellaneous natural products have been shown to protect hepatocytes by enhancing antioxidant defenses and suppressing iron‐dependent cell death. The plant sterol ester of α‐linolenic acid alleviated NAFLD by preventing ferroptosis and reducing oxidative liver injury. It reduced hepatic iron overload, suppressed ROS generation, and lowered lipid peroxidation markers, including MDA and 4‐HNE. Mechanistically, it promoted nuclear translocation of NRF2, leading to upregulation of downstream antioxidant enzymes, including HMOX11 and NAD(P)H:quinone oxidoreductase 1 (NQO1), while strengthening the SLC7A11/GPX4 antioxidant defense system. Concurrently, it suppressed the lipid peroxidation‐associated enzymes ACSL4, arachidonate 5‐lipoxygenase activating protein (ALOX5AP), and PTGS2, thereby interrupting the ferroptotic cascade and preserving hepatocyte viability [169].

Similarly, spermidine, a naturally occurring polyamine, protected against NAFLD through the inhibition of ferroptosis and improvement of hepatic function. Spermidine reduced intracellular iron, MDA, and ROS levels by inducing ATF4, which subsequently increased the expression of SLC7A11, the glutamate‐cysteine ligase modifier subunit (GCLM), and GPX4. Importantly, ATF4 knockdown abolished these protective effects, confirming the central role of the ATF4‐mediated ferroptosis regulatory pathway in spermidine‐induced hepatoprotection [170].

*Hypericum perforatum* L. extract also demonstrated protective effects against NAFLD through coordinated regulation of multiple pathological pathways. The extract reduced hepatic injury, lipid accumulation, and cell death while suppressing inflammatory responses through inhibition of the nuclear factor kappa‐light‐chain‐enhancer of activated B cells (NF‐κB)/PTGS2 signaling pathway. Simultaneously, it alleviated oxidative stress and corrected lipid dysregulation through the activation of the NRF2/PPARα signaling axis. Furthermore, the extract inhibited iron‐dependent ferroptosis through NRF2‐mediated enhancement of antioxidant defenses, highlighting its potential as a multi‐target therapeutic agent for maintaining hepatic homeostasis [171].

The anti‐ferroptotic effects of miscellaneous natural products have also been observed in more advanced chronic liver diseases. Ultrafine garlic powder alleviated both NASH and liver fibrosis by reducing hepatocyte injury, hepatic lipid accumulation, tissue fibrosis, and inflammatory responses while restoring normal liver‐to‐body weight ratios. Mechanistically, this micronized preparation preserved mitochondrial function and maintained iron homeostasis by reducing intracellular ferrous iron (Fe^2+^) levels and suppressing erastin‐induced ferroptosis, thereby protecting the hepatic tissue from progressive damage [172].

In contrast, the induction of ferroptosis appears beneficial in liver cancer. Benja‐ummarit, a traditional Thai herbal formula, suppressed HCC progression by promoting ferroptotic cell death. Treatment induced characteristic ferroptosis‐associated morphological alterations, including cellular ballooning and mitochondrial damage. Mechanistically, Benja‐ummarit disrupted antioxidant defenses through downregulation of NRF2 and GPX4 while simultaneously increasing KEAP1 expression, thereby enhancing cellular susceptibility to ferroptosis and limiting tumor cell survival [173].

**Figure 2:**
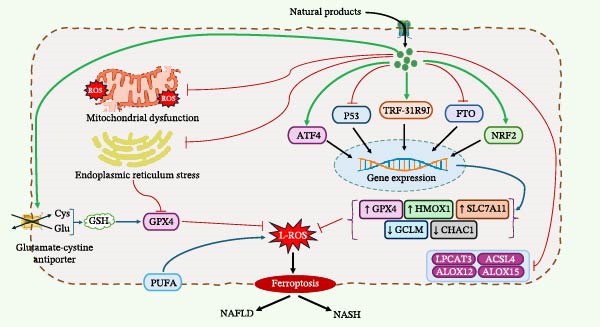
Proposed mechanisms by which natural products modulate ferroptosis in NAFLD and NASH. Natural products regulate ferroptosis‐related signaling pathways by targeting transcription factors such as P53, ATF4, tRF‐31R9J, FTO, and NRF2. These transcriptional regulators modulate the expression of ferroptosis‐associated genes, including GPX4, HMOX1, SLC7A11, GCLM, and CHAC1. Additionally, by alleviating mitochondrial dysfunction and endoplasmic reticulum stress, preserving GSH levels, sustaining GPX4 activity, and reducing L‐ROS accumulation, natural products inhibit ferroptosis and protect against both NAFLD and NASH. ATF4, activating transcription factor 4; CHAC1, ChaC glutathione‐specific γ‐glutamylcyclotransferase 1; FTO, fat mass and obesity‐associated protein; GCLM, glutamate–cysteine ligase modifier subunit; GPX4, glutathione peroxidase 4; GSH, glutathione; HMOX1, heme oxygenase 1; L‐ROS, lipid reactive oxygen species; NAFLD, nonalcoholic fatty liver disease; NASH, nonalcoholic steatohepatitis; NRF2, nuclear factor erythroid 2–related factor 2; SLC7A11, solute carrier family 7 member 11; tRF‐31R9J, tRNA‐derived fragment 31R9J.

**Figure 3:**
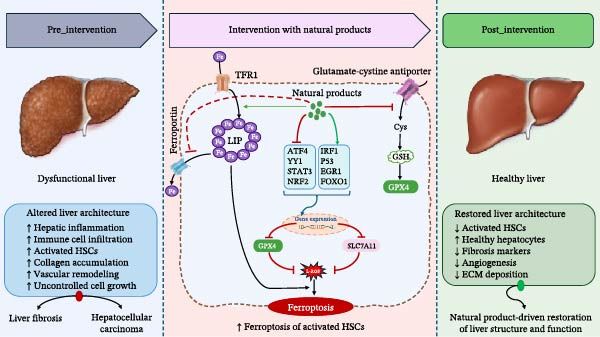
Natural products‐induced ferroptosis in activated HSCs as a mechanism for ameliorating liver fibrosis and HCC. Liver fibrosis and HCC are characterized by altered liver architecture, hepatic inflammation, immune cell infiltration, activation of HSCs, collagen accumulation, vascular remodeling, and uncontrolled cell growth. Natural products target ferroptosis‐related pathways by regulating transcription factors including ATF4, YY1, STAT3, NRF2, IRF1, P53, EGR1, and FOXO1. These regulatory effects downregulate GPX4 and SLC7A11, major inhibitors of ferroptosis, thereby promoting ferroptotic cell death in activated HSCs. Induction of ferroptosis by natural products reduces fibrogenic activity, decreases ECM deposition, and restores liver architecture, ultimately alleviating liver fibrosis and HCC. ATF4, activating transcription factor 4; ECM, extracellular matrix; EGR1, early growth response protein 1; FOXO1, forkhead box protein O1; GPX4, glutathione peroxidase 4; HCC, hepatocellular carcinoma; HSCs, hepatic stellate cells; IRF1, interferon regulatory factor 1; NRF2, nuclear factor erythroid 2–related factor 2; P53, tumor protein p53; SLC7A11, solute carrier family 7 member 11; STAT3, signal transducer and activator of transcription 3; YY1, Yin Yang 1.

Collectively, these findings demonstrate that natural products exert disease‐specific regulation of ferroptosis across chronic liver diseases. In NAFLD and NASH, they predominantly suppress ferroptosis through the activation of antioxidant pathways and restoration of redox homeostasis (see Figure 2). In contrast, during liver fibrosis and HCC, they selectively induce ferroptosis in activated HSCs or malignant hepatocytes, thereby reducing fibrogenesis and tumor progression (see Figure 3). This context‐dependent modulation of ferroptosis highlights the therapeutic versatility of natural products and supports their continued investigation as candidates for chronic liver disease treatment (see Table 2). However, further studies are recommended to highlight the efficacy of these natural products in clinical settings.

**Table 2:** Natural products modulating ferroptosis pathways in chronic liver diseases.

Disease type	Natural product	Study model or cells	Main targets	References
NAFLD	Puerarin	C57BL/6 J mice, AML12 cells	↑ SIRT1, ↑ NRF2, ↑ GPX4	[104]
	Oroxylin A	HFD‐fed C57BL/6J mice; HepG2 cells	↑ NRF2, ↑ GPX4, ↓ KEAP1, ↑ SLC7A11, ↑ HMOX1	[105]
	Hesperetin	Male SD rats, HepG2 cells	↓ hepatic inflammation, ↑ FXR, ↑GCLC, ↑GPX4, ↑ SLC7A11, ↑ SOD, ↓ TNF‐α, ↓IL‐1β, ↓ IL‐6	[106]
	Silymarin	HFD‐induced male C57BL/6J mice, HepG2 cells	↓ lipid accumulation, ↓ MDA, ↓ ROS, ↑NRF2, ↑ SLC7A11, ↑ GPX4	[107]
	Arbutin	C57BL/6 mice, HepG2 cells	↓ FTO, ↑ m6A methylation of SLC7A11, ↑ SLC7A11	[108]
	Kaempferol	HFD‐induced hepatocyte toxicity in AML12 and THLE‐2 cells	↓ SREBP‐1c, ↑SOD, ↑GSH, ↑NRF2, ↑ SOD, ↑ SLC7A11, ↑ GPX4, ↑ AMPK	[109]
	Acacetin	C57BL/6 mice, HepG2 cells	↓ ER stress, ↑ GPX4, ↓ ACSL4	[110]
	EGCG	C57BL/6J mice, L‐02 cells	↑ GPX4, ↑ GSH, ↓ ACSL4, ↓ Mitochondrial ROS, ↓ lipid peroxidation	[111]
	Resveratrol	MCD diet induced C57BL/6 male mice model, HepG2 cells	↑ GSH, ↓ MDA, ↓4‐HNE, ↓ Fe2+, ↑ NRF2, ↑ HMOX1, ↓ ACSL4, ↓ TFR1	[112]
	Ginkgolide B	C57/BL6 ApoE−/−mice, HepG2 cells	↑ NRF2, ↑ GPX4, ↑ HMOX1, ↓ TFR1, ↑ FTH1	[130]
	Diosgenin	HepG2 cells, HFD‐induced rat	↑ NRF2, ↓ ROS, ↓ MDA, ↑ SOD	[131]
	Chryindicolide O	HFD‐ induced zebrafish, AML12 cells	↑ SIRT1, ↑GPX4, ↑ FOXO1 nuclear translocation, ↑ GSH, ↑ SOD	[132]
	Paris saponin VII	HFD‐induced rats and oleic acid‐induced HepG2 cells	↓ hepatic steatosis, ↓ lipid accumulation, ↓ Fe2+, ↓MDA, ↓ROS, ↓TFR1, ↓ ACSL4, ↑ SOD, ↑GSH, ↑SLC7A11, ↑ GPX4, ↑ USP7, ↑ SLC7A11	[133]
	ECGFG	Zebrafish models of NAFLD	↑ SLC7A11, ↓ SREBP1, ↑ FPN, ↑ GPX4, ↑ TFR1	[158]
	Xiaoyao San	HFD‐induced in male Sprague‐Dawley (SD) rats	↓ liver injury, ↑ PPARα, ↑GPX4, ↓ FOXO1, ↓p‐STAT3, ↓HMOX1, ↑ PPARα, ↑ GPX4, ↓ HMOX1	[159]
	Hu Gan Tang	HFD‐induced rat model, HepG2 cells	↑ NRF2, ↑ SLC7A11, ↑GPX4, ↑ FTH1, ↓ TFR1, ↓ ACSL4, ↓ lipid accumulation, ↓ ROS	[160]
	α‐linolenic acid	HFD‐induced in male C57BL/6J mice model, HepG2 cells	↓ TFR1, ↑ FTH1, ↓ROS, ↓ 4‐HNE, ↓ MDA, ↑ GPX4, ↑ SLC7A11, ↑Nrf2, ↓ACSL4, ↓ALOX5, ↓ PTGS2	[169]
	Spermidine	AML‐12 cells	↑ ATF4, ↑ SLC7A11, ↑ GCLM, ↑ GPX4	[170]
	*Hypericum perforatum* L. extract	HFD‐induced model in C57BL/6J mice, AML12 cells	↓ liver steatosis ↑liver function, ↑ NRF2, ↑HMOX1, ↑NQO1, ↓ Fe2^+^ levels, ↑ PPARγ, ↑ GPX4, ↓ ACSL4	[171]
NASH	Icariin	C57BL/6J mice	↑ NRF2, ↑ SLC7A11, ↑ GPX4, ↓ ACSL4, ↓ ALOX12, ↓ mitochondrial ROS	[113]
	Tectorigenin	HepG2 cells, C57BL/6 mice	↑ tRF‐31R9J, ↑ HDAC1 recruitment, ↓ histone lactylation and acetylation on pro‐ferroptosis genes (ATF3, ATF4, CHAC1), ↑ GPX4, ↓ ACSL4	[114]
	Hydroxytyrosol	HFD‐induced C57BL/6J male mice, LX2 cells	↓ Ferroptosis, ↑ FTH1, ↑ FTL, ↓ ACSL4, ↓PTGS2, ↑ SLC7A11, ↑ SOCS2, ↑ GPX4	[115]
	Hinokitiol	High‐fat/high‐cholesterol diet‐fed mice and palmitic acid/oleic acid‐stimulated hepatocytes	↑ NRF2, ↑ GPX4, improved lipid metabolism	[134]
	SGXZ	MCD‐induced NASH mouse model	↑ SLC7A11, ↑ GPX4, ↓ p53, ↓ lipid peroxidation	[161]
	Ultrafine garlic powder	HFD‐induced model in C57BL/6N male mice, LX‐2 cells	↓ ACSL4, ↑ GPX4, ↑ SLC7A11, ↑ FSP1, ↓NCOA, ↓ ROS, ↓ lipid peroxidation	[172]
Liver fibrosis	Ellagic acid	CCl_4_‐induced liver fibrosis in C57BL/6, human LX‐2 cells	↑ ferroptosis, ↓ GSH, ↓ FPN translocation, ↓ VAMP2 (via ↑ proteasomal degradation), impaired VAMP2/SNARE complexes	[116]
	Astragalin	C57BL/6J mice, HSC‐T6 cells, 0.06% thioacetamide treated Zebrafish	↑TFR1, ↑ DMT1, ↑ lipid peroxidation, ↑ PTGS2, ↑ASCL4, ↑ CHAC1, ↓ GPX4	[117]
	Baicalein	CCl_4_‐induced liver injury in C57BL/6 J mice	↑ ferroptosis, ↓ DNMT1, ↓ SCARA5 methylation, ↓ GPX4	[118]
	Danshensu	LPS‐induced LX‐2 and T6 hepatic stellate cells	↑ GPX4, ↑ SLC7A11, ↑ collagen I, ↑ lipid ROS	[119]
	Wogonoside	CCl_4_‐induced liver fibrosis in C57BL/6 mice, HSC‐T6 cells	↑ ferroptosis, ↑ MDA mediated via ↑ SOCS1, ↑ p53, ↓ SLC7A11 axis, ↓ GPX4, ↓ GSH, ↑ iron	[120]
	*Ficus hirta* Vahl.	C57BL/6J mice, HSCs	↑ GSH, ↑ GPX4, ↑ ferroptosis, ↓ ACSL4	[121]
	Formononetin	CCl_4_‐induced hepatic fibrosis in rats, activated HSCs	↑ ferroptosis, ↑ NOX4 activity, ↑ Lipid ROS, ↓ collagen deposition	[122]
	Resveratrol	CCl_4_‐induced fibrotic C57BL/6J mice, LX‐2 cells	↑ ferroptosis (↓ GPX4, ↓ GCLC, ↓ GSH Synthetase (GSS)), ↓ ATF4, ↓ GRP78, ↓ α‐SMA, ↓ Collagen I	[123]
	Rubimaillin	CCl_4_‐induced liver fibrosis model in male C57BL/6J mice, LX2 cells	↑ ferroptosis, ↓ NRF2/GPX4, ↓ HSCs (↓ α‐SMA, ↓ Col1A1), ↓HMOX1, ↓SLC7A11, ↓ GCLC, ↑ ACSL3, ↑ ACSL4, ↑ ACSL5	[124]
	Ginsenoside Rg3	C57BL/6 mice, HSCs	↑ ferroptosis, ↑ ACSL4 (via ↓ methylation), ↓ GSH, ↑ lipid ROS, ↑ iron	[135]
	Dihydrotanshinone	CCl_4_‐induced liver fibrosis in C57BL/6J mice, HSCs	↑ ferroptosis in HSCs, ↑ EGR1, ↓ DNMT1, ↑ ALOX15 (via demethylation), ↑ ROS	[136]
	GAMG	C57BL/6 mice, Inflammatory macrophages	↑ IRF1, ↓ SLC7A11, ↑ ferroptosis, ↓ GSH	[137]
	Ginsenoside Rh2	CCl_4_‐induced liver fibrosis in C57BL/6J mice, primary HSCs	↑ ferroptosis, ↑ IRF1, ↓ SLC7A11, ↓ GSH, ↑ ROS, ↑ iron, ↑ MDA, ↓ HSC activation, ↓ macrophage recruitment	[138]
	Celastrol	CCl_4_‐induced hepatic fibrosis in C57BL/6, activated HSCs	↑ ferroptosis, direct binding to PRDX1/2/4/6 (↓ antioxidant activity), ↑ HMOX1 expression, ↑ lipid peroxidation	[139]
	Ginsenoside Rb1	CCl_4_‐induced liver fibrosis in C57BL/6J mice, primary HSCs, LX‐2 cells	↑ ferroptosis (HSC inactivation mediated via Beclin‐1/SLC7A11 axis), ↑ Beclin‐1, ↓ SLC7A11, ↓ GSH	[140]
	Magnoflorine	CCl_4_‐induced liver fibrosis in C57BL/6J mice; ROS‐activated HSCs in vitro	↑ ferroptosis, ↓ TGF‐β/Smad pathway, ↓ HSC activation markers, ↓ α‐SMA, ↓GPX4, ↓ SLC7A11	[151]
	Berberine	C57BL/6 mice, HSCs	↑ ferroptosis, ↑Fe2+, ↓ GSH, ↑ lipid peroxidation, ↓ GPX4, ↓ FTH1	[152]
	Daphnetin	CCl_4_‐induced liver fibrosis model in male C57BL/6J mice, LX‐2 cells	↑ ferroptosis, ↓ Collagen 1A1, ↓α‐ SMA, ↑ TFR1, ↓ FPN, ↓ GPX4, ↑ NCOA, ↑ Beclin 1, ↓FTH1, ↑ GPX4 Ubiquitination	[154]
	Paeoniae radix alba polysaccharide	CCl_4_‐induced model in male SD rats, erastin‐induced LX‐2 cells	↑ GPX4, ↑ SLC7A1, ↑SOD, ↑GSH, ↑ GSH, ↓ MDA, ↓ROS, ↓ hepatic fibrosis (↓ collagen I, ↓ α‐SMA), ↓ PI3K, ↓PKB, ↓mTOR	[162]
	Albiflorin	CCl_4_‐induced model in male C57BL/6 mice, AML12 mouse hepatocyte line	↑ GSH, ↑ SOD, ↓ MDA, ↓ hepatic injury, ↓ kEAP1, ↑ SLC7A11/GPX4 pathway, ↓ collagen deposition, ↑ FTH1, ↑ NRF2, ↓ TFR1, ↓ HMOX1	[163]
	Longchai decoction	CCl_4_‐induced model in male BALB/c mice, erastin‐induced in LX2 cells	↓ liver injury (↓ Collagen I, ↓ α‐SMA), ↓ inflammation (↓IL‐1β/IL‐10), ↓ lipid peroxidation, ↓ ROS, ↑ NRF2/GPX4, ↑ HMOX1	[164]
	Yindanpinggan capsule	CCl_4_‐induced liver fibrosis model in Male C57BL/6J mice	↑ PPAR γ/GPX4, ↑SLC3A2, ↑SLC7A11, ↑NRF2, ↑HMOX1	[165]
	Taohong Siwu Decoction	CCl_4_‐induced liver injury in C57BL/6 J mice and erastin induced‐BRL‐3A cells	↑ GPX4, ↓ TFR1, ↓ ROS, ↓ MDA, ↑ SLC7A11, ↑ NRF2, ↓ KEAP1	[166]
HCC	Licochalcone A	HepG2 and Huh‐7 cells	↑ ferroptosis, ↓ SLC7A11, ↓ GSH, ↓ GPX4, ↑ ROS →	[125]
	Sappanone A	HCC cell lines, in vivo model	↑ ferroptosis, targets IMPDH2, ↓ NRF2, ↓ GPX4, ↓ SLC7A11	[126]
	Curcumin	C57BL/6J mice, HepG2 cells	↑ ferroptosis, ↓ GPX4, ↓ SLC7A11, ↓ NRF2, ↓ P62, ↑ KEAP1, ↑ ROS, ↑ MDA	[127]
	Emodin	Male BALB/c nude mice, human HCC cell lines HepG2 and MHCC97H	↑ ferroptosis, ↓ GSH, ↑ TFR1, ↓ SLC7A11, ↓GPX4, ↑ ROS, ↓ NFE2L3	[128]
	Macelignan	Female BALB/c nude mice, HCC cell lines, HepG2 cells, Huh‐7 cells	↑ ferroptosis, ↓ M2 polarization of macrophages, ↑TFR1, ↓ SLC7A11, ↓ GPX4	[129]
	Saikosaponin A	HCC cells lines	↑ ferroptosis via ER stress, ↑ ATF3, ↓ SLC7A11, ↓ GSH, ↑MDA, ↑ iron	[141]
	Dihydroartemisinin	HCC cells, subcutaneous xenograft (in vivo/in vitro)	↑ ferroptosis, ↓ ATF4, ↓ SLC7A11	[142]
	SSPH I	HCC cells and xenograft models	↑ ERK1/2, ↓ NRF2/HMOX1 axis, ↑ Iron, ↑ mitochondrial dysfunction	[143]
	Oridonin	HepG2 cells	↑ ferroptosis, ↑ Fe2+, ↑ lipid ROS, ↓SLC7A11, ↓ GPX4, ↑ HMOX1	[144]
	Polyphyllin I	HCC cell lines, nude mice xenograft	↑ ferroptosis, ↓NRF2/HMOX1/GPX4 axis, ↑ Fe2+, ↓ GSH, ↓ SLC7A11, ↓ GPX4	[145]
	Monotropein	HCC cells (in vitro and in vivo tumorigenicity models)	↓ NRF2, ↓ HMOX1, ↓ GPX4, ↑ Fe^2+^, ↑ ROS, ↑ MDA, ↓ GSH, ferroptosis induction linked to anti‐tumor effects	[146]
	Polyphyllin VI	HCCLM3 and Huh7 cells, xenograft model	↑ ferroptosis, ↓ STAT3 phosphorylation, ↓ GPX4	[147]
	Ginsenoside compound K	HepG2 cells, Hep3B cells	↑ ferroptosis, ↑ROS, ↑mitochondrial damage, ↑Fe^2+^, ↓ GPX4	[148]
	Ginsenoside compound k	HepG2, SK‐Hep‐1 cells, HepG2 xenograft in nude mice	↑ ferroptosis, ↓ FOXO1 phosphorylation, ↓ SLC7A11, ↓ GPX4	[149]
	Parthenolide	HepG2, McARH7777 (rat) HCC cells	↑ ferroptosis, ↑ mitochondrial dysfunction, ↑ lipid peroxidation, ↓ GSH, ↓ GPX4	[150]
	Solasonine	HepG2, HepRG cells; mouse xenograft	↑ ferroptosis, ↓ GPX4, ↓ GSH synthetase (GSS)	[153]
	Auraptene	HCC cell lines	↑ ferroptosis, ↑ lipid ROS, ↓ GSH, ↑ SLC7A11 ubiquitin‐proteasomal degradation	[155]
	Esculetin	HCC cells lines	↑ ferroptosis, ↓NRF2/SLC7A11/GPX4 pathway	[156]
	Decursin	Male BALB/c nude mice, Huh7 and HepG2	↓ NRF2, ↓ GPX4, ↓ SLC7A11, ↓ GSH, ↑ MDA, ROS	[157]
	Oxymatrine	HCC cells, xenograft models	↑ ferroptosis, ↑SIRT1, ↓ YY1, ↓ GPX4	[167]
	Hua Zheng San Ji Fang	SK‐HEP‐1 and HepG2 cells	↑ ferroptosis, ↑KEAP1, ↓ NRF2/HMOX1, ↓GSH, ↓ SOD, ↓GPX4, ↓FTH1, ↓ SLC7A11, ↑TFR1	[168]
	Benja‐ummarit	HepG2, HuH‐7, LX‐2 cells, rat liver tissue	↑ ferroptosis, ↑ intracellular iron, ↓ FTH1, ↓ NRF2, ↓ GPX4, ↑ KEAP1	[173]

*Note:* Regulation of ferroptosis pathways by natural products across different liver diseases. this table summarizes the main molecular targets, mechanisms of action, and study models of various natural compounds and herbal formulas modulating ferroptosis in nonalcoholic fatty liver disease (NAFLD), nonalcoholic steatohepatitis (NASH), liver fibrosis, and hepatocellular carcinoma (HCC). Symbols: ↑, Upregulation/Increase; ↓, Downregulation/Decrease.

Abbreviations: 4‐HNE, 4‐hydroxynonenal; 5‐LOX, 5‐lipoxygenase; ACSL3/4/5, acyl‐CoA long‐chain family member 3/4/5; ALF, acute liver failure; ALOX12, arachidonate 12‐lipoxygenase; AMPK, AMP‐activated protein kinase; ATF3/4, activating transcription factor 3/4; CHAC1, ChaC glutathione specific gamma‐glutamylcyclotransferase 1; Col1A1/Collagen I, collagen type I alpha 1 chain; DNMT1, DNA methyltransferase 1; ECGFG, Exocarpium Citri Grandis flavonoids group; EGCG, epigallocatechin gallate; EGR1, early growth response 1; ER, endoplasmic reticulum; ERK1/2, extracellular signal‐regulated kinase 1/2; Fe^2+^, ferrous iron; FOXO1, Forkhead box protein O1; FPN, ferroportin; FSP1, ferroptosis suppressor protein 1; FTH1, ferritin heavy chain 1; FTL, ferritin light chain; FTO, fat mass and obesity‐associated protein; FXR, farnesoid X receptor; GAMG, glycyrrhizic acid alpha‐D‐mono‐glucuronide; GCLC, glutamate‐cysteine ligase catalytic subunit; GCLM, glutamate‐cysteine ligase modifier subunit; GPX4, glutathione peroxidase 4; GRP78, glucose‐regulated protein 78; GSH, glutathione; GSS, glutathione synthetase; HCC, hepatocellular carcinoma; HDAC1, histone deacetylase 1; HFD, high‐fat diet; HMOX1, heme oxygenase 1; HSC, hepatic stellate cell; IL‐1β/6/10, interleukin 1 beta/6/10; IMPDH2, inosine monophosphate dehydrogenase 2; IRF1, interferon regulatory factor 1; KEAP1, Kelch‐like ECH‐associated protein 1; LPS, lipopolysaccharide; m6A, N6‐methyladenosine; MCD, methionine‐choline‐deficient diet; MDA, malondialdehyde; mTOR, mechanistic target of rapamycin; NAFLD, non‐alcoholic fatty liver disease; NASH, non‐alcoholic steatohepatitis; NCOA4, nuclear receptor coactivator 4; NFE2L3, nuclear factor, erythroid 2 like 3; NOX4, NADPH oxidase 4; NQO1, NAD(P)H quinone dehydrogenase 1; NRF2, nuclear factor erythroid 2‐related factor 2; p‐STAT3, phosphorylated signal transducer and activator of transcription 3; PI3K, phosphoinositide 3‐kinase; PKB, protein kinase B; PPARα/γ, peroxisome proliferator‐activated receptor alpha/gamma; PRDX1/2/4/6, peroxiredoxin 1/2/4/6; PTGS2, prostaglandin‐endopeptidase 2; ROS, reactive oxygen species; SCARA5, Scavenger receptor class A member 5; SGXZ, San‐Guo‐Zhi‐Zan formula; SIRT1, sirtuin 1; SLC3A2, solute carrier family 3 member 2; SLC7A11/SLC7A1, solute carrier family 7 member 11/1; Smad, mothers against decapentaplegic homolog; SNARE, soluble N‐ethylmaleimide‐sensitive factor attachment protein receptor; SOCS1/2, suppressor of cytokine signaling 1/2; SOD, superoxide dismutase; SREBP‐1c, sterol regulatory element‐binding protein 1c; STAT3, signal transducer and activator of transcription 3; TFR1, transferrin receptor 1; TGF‐β, transforming growth factor beta; TNF‐α, tumor necrosis factor alpha; tRF, tRNA‐derived fragment; USP7, ubiquitin specific peptidase 7; VAMP2, vesicle‐associated membrane protein 2; YY1, Yin Yang 1; α‐SMA, alpha‐smooth muscle actin.

## 5. Discussion

The liver, central to numerous physiological processes, is highly susceptible to a wide spectrum of acute and chronic diseases, each with distinct etiologies. Despite advances in healthcare, significant gaps remain in their effective management. Ferroptosis, a regulated, iron‐dependent form of cell death driven by lipid peroxidation, has emerged as a promising therapeutic target in various liver disorders.

This review summarizes how natural products modulate ferroptosis across different liver diseases. In ALF, they bolster antioxidant defenses, mainly through NRF2 activation and upregulation of GPX4, SLC7A11, and GSH, while suppressing pro‐ferroptotic enzymes (ACSL4, ALOX12, and ALOX15) and regulating iron metabolism to prevent overload.

In NAFLD and NASH, natural products inhibit ferroptosis via antioxidant activation, iron homeostasis restoration, metabolic regulation, and epigenetic modulation. Key mechanisms include NRF2‐driven expression of GPX4, SLC7A11, and HMOX1; FTO inhibition to promote m6A methylation of SLC7A11; suppression of ACSL4, ALOX12, ALOX15, and LPCAT3; enhancement of SIRT1 activity; alleviation of ER stress; and upregulation of tRF‐31R9J to repress ferroptosis‐promoting genes.

In contrast, in liver fibrosis and HCC, natural products often induce ferroptosis to eliminate pathological cells. This occurs via SLC7A11 inhibition, GPX4 suppression, increased lipid peroxidation (through ACSL4 upregulation and ROS scavenger reduction), HMOX1‐mediated iron release, reduced iron export via FPN inhibition, and enhanced oxidative stress through ERK1/2 activation, KEAP1‐mediated NRF2 suppression, and NOX4‐driven ROS generation.

However, ferroptosis modulation by natural products is not uniformly beneficial and comes with some challenges. Certain compounds can cause hepatotoxicity by promoting ferroptosis in hepatocytes. For instance, matrine, a plant‐derived alkaloid, has been shown to cause liver injury by promoting ferroptosis in hepatocytes. It disrupted the NRF2/GPX4 antioxidant defense system, decreased the expression of GPX4, HMOX1, and SLC7A11, and disturbed iron homeostasis. The injury was ferroptosis‐dependent as it could be attenuated by ferroptosis inhibitors [174]. Similarly, sodium aescinate, a natural plant extract, induced hepatotoxicity by suppressing ATF4 activity, which led to downregulation of SLC7A11, GSH depletion, impaired GPX4 function, and subsequent ferroptosis [175]. Another example is raw Polygonum multiflorum, which elevated serum ALT and AST levels, induced liver tissue necrosis and inflammation, and promoted ROS accumulation while reducing ferroptosis‐resistance proteins such as GPX4, HMOX1, and FTH1. These effects were likewise reversible by ferroptosis inhibition, underscoring the unexpected capacity of these natural products by ferroptosis induction in hepatotoxicity [176].

In addition, natural products may exhibit dual roles in liver fibrosis, with some inducing ferroptosis to eliminate activated fibrogenic cells, while others inhibit ferroptosis to preserve hepatocyte viability. For example, esculin, a coumarin glucoside, alleviated liver fibrosis by inhibiting ferroptosis and enhancing liver function [177]. Likewise, forsythiaside A, a phenylethanoid glycoside, reduced oxidative stress, mitochondrial damage, and ferroptosis in hepatocytes via NRF2 pathway activation [178]. These contrasting outcomes underscore the context‐dependent nature of ferroptosis modulation, which may vary based on the stage or severity of liver disease. It is plausible that in early‐stage fibrosis, natural products exert protective effects by inhibiting ferroptosis, whereas in advanced stages, ferroptosis induction might be beneficial for removing fibrogenic cells.

Furthermore, despite the promising therapeutic implications, translating the findings into clinical practice faces several challenges. First, most available data stem from preclinical models. While these findings are compelling, clinical validation remains lacking. Second, some plant‐derived compounds possess intrinsic hepatotoxic potential, which complicates their widespread therapeutic use. Identifying safe and effective dose ranges is therefore a critical priority to maximize therapeutic benefits while minimizing adverse effects.

Moreover, understanding how ferroptosis modulation affects specific cell types and disease stages is essential. A single compound may confer protection in the early stages of disease yet prove detrimental in advanced fibrosis or cirrhosis, highlighting the need for a nuanced understanding of temporal and cellular specificity.

Furthermore, natural products often exhibit poor solubility and low bioavailability, which limit their therapeutic efficacy. Although several delivery systems have been developed to address these challenges, optimized strategies that effectively translate the beneficial effects of natural products into clinical settings are still lacking [179].

As stated, some challenges related to clinical translation, including poor bio‐availability, unfavorable pharmacokinetics, and standardization hurdles, remain significant. However, natural products hold considerable promise not only as monotherapies but also as adjuvants that enhance the efficacy of existing pharmacological therapies. Recent studies have highlighted their ability to synergize with ferroptosis‐inducing agents in HCC, adding an important translational dimension to their therapeutic value. For instance, naringenin, a natural flavonoid, enhanced the efficacy of ferroptosis inducers including, erastin, RSL3, and sorafenib, by attenuating aerobic glycolysis through activation of the AMPK/PPARγ coactivator 1‐α signaling axis. This metabolic reprogramming led to increased lipid peroxidation and ferroptosis in both liver cancer cells and xenograft models [180].

Similarly, the natural alkaloid 6‐Methoxydihydrosanguinarine induced ferroptosis in HCC cells by downregulating GPX4. Notably, low concentrations of this natural product sensitized HCC cells to ferroptosis inducers such as RSL3 and imidazole ketone erastin, suggesting its synergistic potential when used in combination with other ferroptosis‐associated agents [181].

*Solanum torvum*, a traditional medicinal plant, induced ferroptosis in HCC cells through GPX4 downregulation and HMOX1 upregulation. Importantly, *S. torvum* significantly enhanced the anticancer efficacy of lenvatinib in both lenvatinib‐sensitive and resistant cell lines, suggesting a role in overcoming drug resistance and reinforcing ferroptosis pathways [182].

Furthermore, Iberverin, a natural product isolated from *Brassica oleracea* var. capitata, exerted anti‐tumor effects in HCC by inducing ferroptosis through the downregulation of SLC7A11 and promotion of ubiquitin‐dependent GPX4 degradation. It also sensitized HCC cells to RSL3 and the imidazole ketone erastin, supporting its use in both monotherapy and combination approaches targeting ferroptosis [183].

These findings underscore the promising role of plant‐derived natural products in potentiating ferroptosis‐based therapies. Through various mechanisms, these compounds not only enhance the efficacy of existing ferroptosis inducers but also help overcome chemoresistance in liver cancer treatment. Therefore, their integration into combination regimens could offer a compelling avenue for improved therapeutic strategies in HCC.

However, robust preclinical and clinical investigations are still required to establish both the safety and efficacy of these compounds in human populations. Only through well‐designed trials can these mechanistic insights be translated into effective, evidence‐based therapies for liver diseases.

## 6. Concluding Remarks

Taken together, these findings demonstrate that plant‐derived natural products hold substantial promise for mitigating liver diseases through multi‐target modulation of ferroptosis and associated molecular pathways. This review integrates a comprehensive literature survey with detailed mechanistic analysis, offering direct evidence of ferroptosis regulation in diverse hepatic conditions. By mapping upstream regulators, identifying key ferroptosis mediators, and clarifying critical signaling networks, it provides a robust framework to guide future research.

## Author Contributions

**Farshad Niazpour:** conceptualization, data curation, formal analysis, investigation, methodology, project administration, validation, visualization, writing – original draft, writing – review and editing. **Reza Meshkani:** resources, supervision, validation, visualization, writing – original draft, writing – review and editing.

## Funding

The authors declare no specific funding for this work.

## Conflicts of Interest

The authors declare no conflicts of interest.

## Data Availability

Data sharing is not applicable to this article as no datasets were generated or analyzed during the current study.

## References

[1] Fernández J. , Bassegoda O. , Toapanta D. , and Bernal W. , Acute liver failure: A practical update, JHEP Reports. (2024) 6, no. 9, 10.1016/j.jhepr.2024.101131, 101131. 39170946 PMC11337735

[2] Jamioł-Milc D. , Gudan A. , and Kaźmierczak-Siedlecka K. , et al.Nutritional Support for Liver Diseases, Nutrients. (2023) 15, no. 16, 10.3390/nu15163640, 3640. 37630830 PMC10459677

[3] Ghazanfar H. , Javed N. , and Qasim A. , et al.Metabolic Dysfunction-Associated Steatohepatitis and Progression to Hepatocellular Carcinoma: A Literature Review, Cancers. (2024) 16, no. 6, 10.3390/cancers16061214, 1214. 38539547 PMC10969013

[4] Samy A. M. , Kandeil M. A. , Sabry D. , Abdel-Ghany A. A. , and Mahmoud M. O. , From NAFLD to NASH: Understanding the Spectrum of Non-Alcoholic Liver Diseases and Their Consequences, Heliyon. (2024) 10, no. 9, 10.1016/j.heliyon.2024.e30387, e30387. 38737288 PMC11088336

[5] Kim H. Y. , Yu J. H. , and Chon Y. E. , et al.D.W.: Prevalence of Clinically Significant Liver Fibrosis in the General Population: A Systematic Review and Meta-Analysis, Clinical and Molecular Hepatology. (2024) 30, no. Suppl, S199–S213, 10.3350/cmh.2024.0351. 39074982 PMC11493351

[6] Li C. , Lu B. , and Deng B. , New Insights Into the Diagnosis and Treatment of Hepatocellular Carcinoma, Biomedicines. (2025) 13, no. 5, 10.3390/biomedicines13051244, 1244. 40427070 PMC12109435

[7] Zou P. , He Q. , Xia H. , and Zhong W. , Ferroptosis and Its Impact on Common Diseases, PeerJ. (2024) 12, 10.7717/peerj.18708, e18708. 39713140 PMC11663406

[8] Deng L. , Tian W. , and Luo L. , Application of Natural Products in Regulating Ferroptosis in Human Diseases, Phytomedicine. (2024) 128, 10.1016/j.phymed.2024.155384, 155384. 38547620

[9] Xie Y. , Hou W. , and Song X. , et al.Ferroptosis: Process and Function, Cell Death & Differentiation. (2016) 23, no. 3, 369–379, 10.1038/cdd.2015.158. 26794443 PMC5072448

[10] Hemmati N. , Anoush M. , Kiasari B. A. , and Torkamani A. , Targeting Ferroptosis as a Therapeutic Strategy for Hepatotoxicity, Toxicology Reports. (2025) 15, 10.1016/j.toxrep.2025.102137, 102137. 41140341 PMC12549406

[11] Yang X. , Cui H. , and Zhang R. , et al.Role of Traditional Chinese Medicine in Ferroptosis of Liver Cancer: Focus on Signaling Pathways, Phytomedicine. (2026) 155, 10.1016/j.phymed.2026.158106, 158106. 41932001

[12] Loomba R. , Wong R. , and Fraysse J. , et al.Nonalcoholic Fatty Liver Disease Progression Rates to Cirrhosis and Progression of Cirrhosis to Decompensation and Mortality: A Real World Analysis of Medicare Data, Alimentary Pharmacology & Therapeutics. (2020) 51, no. 11, 1149–1159, 10.1111/apt.15679. 32372515

[13] Dixon S. J. , Lemberg K. M. , and Lamprecht M. R. , et al.Ferroptosis: An Iron-Dependent Form of Nonapoptotic Cell Death, Cell. (2012) 149, no. 5, 1060–1072, 10.1016/j.cell.2012.03.042. 22632970 PMC3367386

[14] Živanović N. , Lesjak M. , Simin Nša , and Srai S. K. S. , Beyond Mortality: Exploring the Influence of Plant Phenolics on Modulating Ferroptosis—A Systematic Review, Antioxidants. (2024) 13, no. 3, 10.3390/antiox13030334, 334. 38539867 PMC10968245

[15] Niazpour F. and Meshkani R. , Unlocking the Therapeutic Potential of Autophagy Modulation by Natural Products in Tackling Non-Alcoholic Fatty Liver Disease, Phytotherapy Research. (2025) 39, no. 5, 2357–2373, 10.1002/ptr.8463. 40184168

[16] Shi J. , Lu Y. , and Wei W. , et al.Ferroptosis: A Novel Pharmacological Mechanism Against Multiple Myeloma, Frontiers in Pharmacology. (2025) 16, 10.3389/fphar.2025.1606804, 1606804. 40735482 PMC12306488

[17] Pizzimenti S. , Ciamporcero E. , and Daga M. , et al.Interaction of Aldehydes Derived From Lipid Peroxidation and Membrane Proteins, Frontiers in Physiology. (2013) 4, 10.3389/fphys.2013.00242, 242. 24027536 PMC3761222

[18] Do Q. and Xu L. , How Do Different Lipid Peroxidation Mechanisms Contribute to Ferroptosis?, Cell Reports Physical Science. (2023) 4, no. 12, 10.1016/j.xcrp.2023.101683, 101683. 38322411 PMC10846681

[19] Niazpour F. and Meshkani R. , Natural Products Targeting Ferroptosis in Type 2 Diabetes Mellitus and Its Complications, Phytotherapy Research. (2025) 39, no. 10, 4658–4676, 10.1002/ptr.70083. 40865926

[20] Zhao N. , Li S. , and Wu H. , et al.Ferroptosis: An Energetic Villain of Age-Related Macular Degeneration, Biomedicines. (2025) 13, no. 4, 10.3390/biomedicines13040986, 986. 40299661 PMC12024642

[21] Fu C. , Cao N. , and Zeng S. , et al.Role of Mitochondria in the Regulation of Ferroptosis and Disease, Frontiers in Medicine. (2023) 10, 10.3389/fmed.2023.1301822, 1301822. 38155662 PMC10753798

[22] Chen X. , Li J. , Kang R. , Klionsky D. J. , and Tang D. , Ferroptosis: Machinery and Regulation, Autophagy. (2021) 17, no. 9, 2054–2081, 10.1080/15548627.2020.1810918. 32804006 PMC8496712

[23] Li W. , Liang L. , Liu S. , Yi H. , and Zhou Y. , FSP1: A Key Regulator of Ferroptosis, Trends in Molecular Medicine. (2023) 29, no. 9, 753–764, 10.1016/j.molmed.2023.05.013. 37357101

[24] Niazpour F. , Hoseini H. , Seyyedebrahimi S. S. , Esfahani E. N. , and Meshkani R. , The Relationship Between Nrf2/Keap1 System and Endoplasmic Reticulum Stress and Inflammatory Markers in Peripheral Blood Mononuclear Cells of type 2 diabetic Subjects, Clinical Diabetology. (2021) 10, no. 5, 394–402, 10.5603/DK.a2021.0032.

[25] Ma Y. , Zhang D. , and Li Z. , Dual Role of Heme Oxygenase-1 in Disease Progression and Treatment: A Literature Review, International Journal of Biological Macromolecules. (2025) 321, 10.1016/j.ijbiomac.2025.146272, 146272. 40712871

[26] Jiang L. , Kon N. , and Li T. , et al.Ferroptosis as a p53-Mediated Activity During Tumour Suppression, Nature. (2015) 520, no. 7545, 57–62, 10.1038/nature14344. 25799988 PMC4455927

[27] Sun Y.-W. , Zhao B.-W. , Li H.-F. , and Zhang G.-X. , Overview of Ferroptosis and Pyroptosis in Acute Liver Failure, World Journal of Gastroenterology. (2024) 30, no. 34, 3856–3861, 10.3748/wjg.v30.i34.3856. 39350783 PMC11438646

[28] Lee W. M. , Squires R. H. , Nyberg S. L. , Doo E. , and Hoofnagle J. H. , Acute Liver Failure: Summary of a Workshop, Hepatology. (2008) 47, no. 4, 1401–1415, 10.1002/hep.22177. 18318440 PMC3381946

[29] Trautwein C. and Koch A. , Gershwin M. E. , Vierling J. M. , and Manns M. P. , Mechanisms of Acute Liver Failure, Liver Immunology, 2014, Springer International Publishing, 373–388.

[30] Patterson J. , Hussey H. S. , and Silal S. , et al.Systematic Review of the Global Epidemiology of Viral-Induced Acute Liver Failure, BMJ Open. (2020) 10, no. 7, 10.1136/bmjopen-2020-037473, e037473. PMC7375632 32690747

[31] David S. and Hamilton J. P. , Drug-Induced Liver Injury, US Gastroenterology & Hepatology Review. (2010) 6, 73–80. 21874146 PMC3160634

[32] Kang J. , Park S. H. , Khanam M. , Park S. B. , Shin S. , and Seo W. , Impact of Binge Drinking on Alcoholic Liver Disease, Archives of Pharmacal Research. (2025) 48, no. 3, 212–223, 10.1007/s12272-025-01537-1. 40035998

[33] Ha Y. , Jeong I. , and Kim T. H. , Alcohol-Related Liver Disease: An Overview on Pathophysiology, Diagnosis and Therapeutic Perspectives, Biomedicines. (2022) 10, no. 10, 10.3390/biomedicines10102530, 2530. 36289791 PMC9599689

[34] Zhou X.-N. , Zhang Q. , and Peng H. , et al.Silent Information Regulator sirtuin 1 Ameliorates Acute Liver Failure via the p53/Glutathione Peroxidase 4/Gasdermin D Axis, World Journal of Gastroenterology. (2024) 30, no. 11, 1588–1608, 10.3748/wjg.v30.i11.1588. 38617450 PMC11008418

[35] Liu G.-Z. , Xu X.-W. , Tao S.-H. , Gao M.-J. , and Hou Z.-H. , HBx Facilitates Ferroptosis in Acute Liver Failure Via EZH2 Mediated SLC7A11 Suppression, Journal of Biomedical Science. (2021) 28, no. 1, 10.1186/s12929-021-00762-2, 67. 34615538 PMC8495979

[36] Yamada N. , Karasawa T. , and Wakiya T. , et al.Iron Overload as a Risk Factor for Hepatic Ischemia-Reperfusion Injury in Liver Transplantation: Potential Role of Ferroptosis, American Journal of Transplantation. (2020) 20, no. 6, 1606–1618, 10.1111/ajt.15773. 31909544

[37] Du Y. and Guo Z. , Recent Progress in Ferroptosis: Inducers and Inhibitors, Cell Death Discovery. (2022) 8, no. 1, 10.1038/s41420-022-01297-7, 501. 36581640 PMC9800531

[38] Atanasov A. G. , Zotchev S. B. , and Dirsch V. M. , Natural Products in Drug Discovery: Advances and Opportunities, Nature Reviews Drug Discovery. (2021) 20, no. 3, 200–216, 10.1038/s41573-020-00114-z. 33510482 PMC7841765

[39] Rana A. , Samtiya M. , Dhewa T. , Mishra V. , and Aluko R. E. , Health Benefits of Polyphenols: A Concise Review, Journal of Food Biochemistry. (2022) 46, no. 10, 10.1111/jfbc.14264, e14264. 35694805

[40] Tan H. , Ji Y. , and Cheng X. , et al.Chlorogenic Acid Alleviates Chronic Restraint Stress-Induced Liver Injury Potentially *via* Nrf2-Mediated Inhibition of Ferroptosis and Mitochondrial Apoptosis, Food & Function. (2026) 17, no. 2, 1061–1073, 10.1039/D5FO04627K. 41524429

[41] Lin H. , Guo X. , and Liu J. , et al.Ethanol-Induced Hepatic Ferroptosis Is Mediated by PERK-Dependent MAMs Formation: Preventive Role of Quercetin, Molecular Nutrition & Food Research. (2024) 68, no. 7, 10.1002/mnfr.202300343, 2300343. 38501770

[42] Zhang Y. , Dong R. , and Zhou H. , et al.The Flavonoid Glycoside From, *Abrus Cantoniensis*, Hance Alleviates Alcoholic Liver Injury by Inhibiting Ferroptosis in an AMPK-Dependent Manner, Journal of Agricultural and Food Chemistry. (2024) 72, no. 29, 16323–16333, 10.1021/acs.jafc.4c02912. 38990278

[43] Su Y. , Zeng Y. , and Zhou M. , et al.Natural Polyphenol-Mediated Inhibition of Ferroptosis Alleviates Oxidative Damage and Inflammation in Acute Liver Injury, Biomaterials Research. (2025) 29, 10.34133/bmr.0167, 0167. 40103575 PMC11913781

[44] Pei X. , Mu R. , and Liu S. , et al.7,8-Dihydroxyflavone Protects Acetaminophen Induced Liver Injury Through Activating PI3K/Akt/NRF2/GPX4 Mediated Ferroptosis Suppression, Free Radical Biology and Medicine. (2026) 242, 275–287, 10.1016/j.freeradbiomed.2025.10.297. 41173313

[45] Ren J. , Wang Z. , and Zhang Y. , et al.Sesamin Alleviates Drug-Induced Hepatotoxicity via Autophagy Enhancement and Ferroptosis Inhibition, Redox Report. (2025) 30, no. 1, 10.1080/13510002.2025.2588863, 2588863. 41254937 PMC12632232

[46] Pu R. , Cui Q. , Xing Z. , Liu L. , Xiao W. , and Xiao Q. , Scutellarin Modulates Nrf2 to Alleviate Inflammation, Pyroptosis, and Ferroptosis in Acetaminophen-Induced Hepatotoxicity, Phytomedicine. (2026) 155, 10.1016/j.phymed.2026.158107, 158107. 41966027

[47] Long Z. , Yu X. , and Li S. , et al.Sakuranetin Prevents Acetaminophen-Induced Liver Injury via Nrf2-Induced Inhibition of Hepatocyte Ferroptosis, Drug Design, Development and Therapy. (2025) 19, 159–171, 10.2147/DDDT.S497817. 39816848 PMC11733203

[48] Li H. , Weng Q. , and Gong S. , et al.Kaempferol Prevents Acetaminophen-Induced Liver Injury by Suppressing Hepatocyte Ferroptosis *via* Nrf2 Pathway Activation, Food & Function. (2023) 14, no. 4, 1884–1896, 10.1039/D2FO02716J. 36723004

[49] Wu L. , Lv L. , and Xiang Y. , et al.Rosmarinic Acid Protects Against Acetaminophen-Induced Hepatotoxicity by Suppressing Ferroptosis and Oxidative Stress Through Nrf2/HO-1 Activation in Mice, Marine Drugs. (2025) 23, no. 7, 10.3390/md23070287, 287. 40710512 PMC12299429

[50] Han Z. , Batudeligen , and Chen H. , et al.Luteolin Attenuates CCl4-Induced Hepatic Injury by Inhibiting Ferroptosis via SLC7A11, BMC Complementary Medicine and Therapies. (2024) 24, no. 1, 10.1186/s12906-024-04486-2, 193. 38755566 PMC11100030

[51] Yu Y. , Liang J. , and Yuan Z. , et al.Bioactive Compound Schaftoside From *Clinacanthus nutans* Attenuates Acute Liver Injury by Inhibiting Ferroptosis Through Activation the Nrf2/GPX4 Pathway, Journal of Ethnopharmacology. (2024) 328, 10.1016/j.jep.2024.118135, 118135. 38556139

[52] Zhang W. , Liu Z. , and Dong C. , et al.Curcumin Alleviates Ferroptosis in CCl_4_-Induced Acute Liver Injury In Vitro: Involvement of TXNIP/NLRP3 Inhibition and SLC7A11/GSH/GPX4 Activation, Naunyn-Schmiedeberg’s Archives of Pharmacology. (2026) 399, no. 4, 4919–4932, 10.1007/s00210-025-04748-x. 41136720

[53] Chen L. , Kong X. , and Zhou R. , et al.Proteomics Reveals the Pharmacological Mechanism of Flavonoids From Astragali Complanati Semen in Preventing Chronic Liver Injury, Phytomedicine. (2024) 133, 10.1016/j.phymed.2024.155910, 155910. 39059265

[54] Gan J. , Xu T. , and Zhang J. , et al.Sepsis-Induced Liver Injury Mitigated by Isoferulic Acid Through Inhibition of Hepatic Ferroptosis via the SIRT1 Signaling Pathway, Phytotherapy Research. (2026) 40, no. 1, 3–20, 10.1002/ptr.70098. 40968354

[55] Wang W. , Li H. , and Wang Y. , et al.Ferulic Acid Protects Against LPS-Induced Sheep Hepatocytes Oxidative Damage via Activating the GSH-GPX4 Pathway and Inhibiting Lipid Metabolism-Mediated Ferroptosis, Antioxidants. (2025) 14, no. 10, 10.3390/antiox14101185, 1185. 41154494 PMC12562129

[56] Liu Y. , Zhao J. , Cong W. , Liu T. , and Sha K. , Alpinetin Pretreatment Prevents Lipopolysaccharide/D-Galactosamine-Induced Acute Liver Injury in Mice by Inhibiting Ferroptosis via the Nrf2/SLC7A11/GPX4 Pathway, Scientific Reports. (2025) 15, no. 1, 10.1038/s41598-025-26588-8, 40065. 41249398 PMC12623886

[57] Zhou J. , Li Y. , Xi C. , Pan W. , Yu Q. , and Wu Z. , Apigenin Alleviates Fumonisin B1-Induced Hepatotoxicity by Suppressing Ferroptosis Through the Nrf2/FSP1 Pathway, Journal of Agricultural and Food Chemistry. (2025) 73, no. 42, 26999–27011, 10.1021/acs.jafc.5c08870. 41073359

[58] Zhang H.-J. , Luo J.-Z. , and Lan C. , et al.Baicalin Protects Against Hepatocyte Injury Caused by Aflatoxin B1 via the TP53-Related Ferroptosis Pathway, Ecotoxicology and Environmental Safety. (2024) 281, 10.1016/j.ecoenv.2024.116661, 116661. 38954907

[59] Wu L. , Dong B. , and Chen Q. , et al.Effects of Curcumin on Oxidative Stress and Ferroptosis in Acute Ammonia Stress-Induced Liver Injury in Gibel Carp (*Carassius gibelio*), International Journal of Molecular Sciences. (2023) 24, no. 7, 10.3390/ijms24076441, 6441. 37047414 PMC10094298

[60] Jin L. , Zhang Y. , and Xia Y. , et al.Polybrominated Biphenyls Induce Liver Injury by Disrupting the KEAP1/Nrf2/SLC7A11 Axis Leading to Impaired GSH Synthesis and Ferroptosis in Hepatocytes, Archives of Toxicology. (2025) 99, no. 4, 1545–1559, 10.1007/s00204-025-03973-w. 39934342

[61] Wang P. , Yao Q. , and Zhu D. , et al.Resveratrol Protects Against Deoxynivalenol-Induced Ferroptosis in HepG2 Cells, Toxicology. (2023) 494, 10.1016/j.tox.2023.153589, 153589. 37419272

[62] Zhang X. , Zhang F. , and Li M. , et al.Epicatechin Attenuates Emamectin Benzoate-Induced Liver Injury in Grass Carp by Activating Nrf2/GPX4 Signaling Pathway, Fish & Shellfish Immunology. (2025) 157, 10.1016/j.fsi.2025.110118, 110118. 39809417

[63] Lang Y. , Gao N. , and Tan H. , et al.Blueberry Anthocyanins and Their Metabolites Synergistically Alleviated Ferroptosis Induced by Acrylamide in HepG2 Cells, Food Chemistry. (2025) 496, 10.1016/j.foodchem.2025.146690, 146690. 41109184

[64] Liu J. , Li K. , Li S. , Yang G. , Lin Z. , and Miao Z. , Grape Seed-Derived Procyanidin Inhibits Glyphosate-Induced Hepatocyte Ferroptosis via Enhancing Crosstalk Between Nrf2 and FGF12, Phytomedicine. (2024) 123, 10.1016/j.phymed.2023.155278, 155278. 38103315

[65] Fu Y. , Hou L. , Wang S. , Hu H. , and Yin S. , 1,2,3,4,6-Pentagalloyl Glucose Efficiently Chelate Excess Iron to Treat Iron Overload Disorders and to Prevent Ferroptosis, Food Research International. (2026) 227, 10.1016/j.foodres.2025.118229, 118229. 41652759

[66] Fu H. , Li W. , and Cheng Q. , et al.Ellagic Acid Alleviates Iron Overload-Induced Liver Damage by Mitigating Ferroptosis Through Modulation of the TGFβ/Smad Signaling Pathway, Food Research International. (2025) 214, 10.1016/j.foodres.2025.116590, 116590. 40467263

[67] Li M. , Mao Z. , and Lu L. , et al.Licochalcone a Promotes SP1/DNA-PKcs-Dependent NHEJ Repair to Prevent Ferroptosis and Attenuate Hepatic Ischemia-Reperfusion Injury, Phytomedicine. (2026) 150, 10.1016/j.phymed.2025.157650, 157650. 41365195

[68] Nguyen H. N. , Ullevig S. L. , Short J. D. , Wang L. , Ahn Y. J. , and Asmis R. , Ursolic Acid and Related Analogues: Triterpenoids With Broad Health Benefits, Antioxidants. (2021) 10, no. 8, 10.3390/antiox10081161, 1161. 34439409 PMC8388988

[69] Zhou L. , Xiao M. , Li Y. , Chitrakar B. , Sheng Q. , and Zhao W. , Ursolic Acid Ameliorates Alcoholic Liver Injury Through Attenuating Oxidative Stress-Mediated Ferroptosis and Modulating Gut Microbiota, Journal of Agricultural and Food Chemistry. (2024) 72, no. 38, 21181–21192, 10.1021/acs.jafc.4c04762. 39277869

[70] Kannampuzha S. and Gopalakrishnan A. V. , Protective Role of Ursolic Acid Against Cisplatin-Induced Oxidative Stress and Ferroptosis in the Liver of Swiss Albino Mice, Medical Oncology. (2025) 42, no. 8, 10.1007/s12032-025-02854-7, 327. 40652144

[71] Li Y. , Yu P. , and Fu W. , et al.Ginsenoside Rd Inhibited Ferroptosis to Alleviate CCl _4_ -Induced Acute Liver Injury in Mice via cGAS/STING Pathway, The American Journal of Chinese Medicine. (2023) 51, no. 1, 91–105, 10.1142/S0192415X23500064. 36437551

[72] Shan G.-Y. , Shi Y.-P. , Zhang Y.-X. , Wan H. , Gao Z.-C. , and Li H.-J. , Ginsenoside Rg5 Ameliorates Lipopolysaccharide (LPS)-Induced Acute Liver Injury via Interfering Autophagy/Nrf2/Ferroptosis Signal Axis, Phytomedicine. (2025) 144, 10.1016/j.phymed.2025.156941, 156941. 40516288

[73] Ouyang C. , Ma X. , and Zhao J. , et al.Oleanolic Acid Inhibits Mercury Chloride Induced-Liver Ferroptosis by Regulating ROS/Iron Overload, Ecotoxicology and Environmental Safety. (2023) 258, 10.1016/j.ecoenv.2023.114973, 114973. 37163906

[74] Liu X. , Xu Y. , and Yu X. , et al.Saikosaponin D Alleviates Liver Ischemia-Reperfusion Injury by Inhibiting Mitophagy-Associated Ferroptosis via the STAT3/PINK1 Pathway, Phytotherapy Research. (2026) 40, no. 6, 3722–3740, 10.1002/ptr.70333. 41968766

[75] Rampogu S. , Balasubramaniyam T. , and Lee J.-H. , Phytotherapeutic Applications of Alkaloids in Treating Breast Cancer, Biomedicine & Pharmacotherapy. (2022) 155, 10.1016/j.biopha.2022.113760, 113760. 36271547

[76] Tao M. , Shi J. , and Zhang Y. , et al.Berbamine Attenuates Acetaminophen-Induced Liver Injury by Engaging GCLC and Enhancing Ferroptosis-Regulatory Antioxidant Pathways, Biochimica et Biophysica Acta (BBA) - Molecular Basis of Disease. (2026) 1872, no. 4, 10.1016/j.bbadis.2026.168154, 168154. 41539206

[77] Wang M. , Liu C.-Y. , and Wang T. , et al.(+)-Clausenamide Protects Against Drug-Induced Liver Injury by Inhibiting Hepatocyte Ferroptosis, Cell Death & Disease. (2020) 11, 10.1038/s41419-020-02961-5, 781. 32951003 PMC7502081

[78] Xu Y. , Zeng Q. , and Zhang A. , Assessing the Mechanisms and Adjunctive Therapy for Arsenic-Induced Liver Injury in Rats, Environmental Toxicology. (2024) 39, no. 3, 1197–1209, 10.1002/tox.24008. 37902164

[79] Zhang G. , Li J. , and Xu Z. , et al.Exocarpium Citri Grandis Attenuates Lipopolysaccharide-Induced Acute Liver Injury Through Suppression of Inflammatory, Apoptotic, Oxidative, and Ferroptotic Pathways, Food Science & Nutrition. (2025) 13, no. 10, 10.1002/fsn3.71012, e71012. 41019187 PMC12474559

[80] Lin Y. , Du Y. , and Wang M. , et al.Jiedu Huayu Suppress Ferroptosis Induced by 5-Lipoxygenase-Dependent Peroxidation in Acute Liver Failure, Journal of Ethnopharmacology. (2025) 353, 10.1016/j.jep.2025.120343, 120343. 40754030

[81] Lu Y. , Sun H. , and Zhu Q. , et al.Niujiaodihuang Detoxify Decoction Inhibits D-GalN/LPS-Induced Hepatocyte Ferroptosis by Maintaining Mitochondrial Homeostasis via OMA1-OPA1 Pathway, Journal of Physiological Investigation. (2025) 68, no. 5, 286–296, 10.4103/ejpi.EJPI-D-24-00104. 40908818

[82] Zhang Y. , Lu Q. , and Yan Y. , et al.Artemisia Keiskeana Miq. Alleviates Oxidative Stress and Ferroptosis in APAP-Induced Liver Injury by Mediating Nrf2/GPX4/NF-κB Signaling Pathway Through ESR1, Phytomedicine. (2025) 148, 10.1016/j.phymed.2025.157436, 157436. 41151450

[83] Dong Z. , Zhang Z. , and Peng Y. , et al.Pien Tze Huang Ameliorates Alcohol-Associated Liver Disease via Suppressing Oxidative Stress and Ferroptosis, Phytomedicine. (2026) 155, 10.1016/j.phymed.2026.158170, 158170. 41980338

[84] Wei Y.-Y. , Wang H.-R. , and Fan Y.-M. , et al.Acute Liver Injury Induced by Carbon Tetrachloride Reversal by Gandankang Aqueous Extracts Through Nuclear Factor Erythroid 2-Related Factor 2 Signaling Pathway, Ecotoxicology and Environmental Safety. (2023) 251, 10.1016/j.ecoenv.2023.114527, 114527. 36628874

[85] Wei Y. , Gao C. , and Wang H. , et al.Mori Fructus Aqueous Extracts Attenuates Liver Injury by Inhibiting Ferroptosis via the Nrf2 Pathway, Journal of Animal Science and Biotechnology. (2023) 14, no. 1, 10.1186/s40104-023-00845-0, 56. 37032323 PMC10084661

[86] Du H. , Yang K. , and Yang J. , et al. *Euphorbia humifusa* Willd. ex Schltdl. Mitigates Liver Injury via KEAP1-NFE2L2-Mediated Ferroptosis Regulation: Network Pharmacology and Experimental Validation, Veterinary Sciences. (2025) 12, no. 4, 10.3390/vetsci12040350, 350. 40284852 PMC12030869

[87] Zhang L. , Shi W.-Y. , and Xu J.-Y. , et al.Protective Effects and Mechanism of Chemical- and Plant-Based Selenocystine Against Cadmium-Induced Liver Damage, Journal of Hazardous Materials. (2024) 468, 10.1016/j.jhazmat.2024.133812, 133812. 38368684

[88] Jian P.-A. , Yang T.-N. , and Wang Y.-X. , et al.Lycopene, a Natural Plant Extract, Alleviates Atrazine-Induced Ferroptosis in Hepatocytes by Activating Cytochrome P450 Oxidoreductase, International Journal of Biological Macromolecules. (2025) 308, 10.1016/j.ijbiomac.2025.142311, 142311. 40139611

[89] Song C. , Li X. , and Zhang A. , et al.Lycopene Inhibits Zearalenone-Induced Ferroptosis via Activating AMPK/Nrf2 Signaling Pathway in AML-12 Hepatocytes and in Mice Livers, Ecotoxicology and Environmental Safety. (2025) 303, 10.1016/j.ecoenv.2025.119045, 119045. 40945091

[90] Yang X. , Song W. , and Lu Z. , et al.Lycopene Mitigates T-2 Toxin-Induced Hepatic Ferroptosis by Targeting the Nrf2/Mitophagy Axis in Mice, npj Science of Food. (2026) 10, no. 1, 10.1038/s41538-026-00736-4, 94. 41654705 PMC12996414

[91] Zhang Y.-J. , Cheng D. , and Yao W.-H. , et al.Spermidine Alleviates Ethanol-Induced Hepatocyte Ferroptosis by Restoring Hepatic Iron Homeostasis via the Gut-Liver Axis, Phytomedicine. (2026) 152, 10.1016/j.phymed.2026.157853, 157853. 41579592

[92] Zhao Y. , Liu X. , and Liang C. , et al.α-Lipoic Acid Alleviated Fluoride-Induced Hepatocyte Injury via Inhibiting Ferroptosis, Journal of Agricultural and Food Chemistry. (2022) 70, no. 50, 15962–15971, 10.1021/acs.jafc.2c07484. 36459405

[93] Alsirhani A. M. , Abu-Almakarem A. S. , and Alwaili M. A. , et al. *Syzygium aromaticum* Extract Mitigates Doxorubicin-Induced Hepatotoxicity in Male Rats, International Journal of Molecular Sciences. (2024) 25, no. 23, 10.3390/ijms252312541, 12541. 39684253 PMC11641798

[94] Gupta P. , Non-Alcohlic Fatty Liver Disease (NAFLD): Is It a Dormant Volcano or Tip of an Iceberg?, Indian Journal of Community Medicine. (2024) 49, no. 6, 780–785, 10.4103/ijcm.ijcm_174_24. 39668912 PMC11633275

[95] Luo S. , Lu Z. , Wang L. , Li Y. , Zeng Y. , and Lu H. , Hepatocyte HIF-2α Aggravates NAFLD by Inducing Ferroptosis Through Increasing Extracellular Iron, American Journal of Physiology-Endocrinology and Metabolism. (2025) 328, no. 1, E92–E104, 10.1152/ajpendo.00287.2023. 39679942

[96] Noureddin M. and Sanyal A. J. , Pathogenesis of NASH: the Impact of Multiple Pathways, Current Hepatology Reports. (2018) 17, no. 4, 350–360, 10.1007/s11901-018-0425-7. 31380156 PMC6677539

[97] Shu Y.-Y. , Gao W.-K. , Chu H.-K. , Yang L. , Pan X.-L. , and Ye J. , Attenuation by Time-Restricted Feeding of High-Fat and High-Fructose Diet-Induced NASH in Mice Is Related to Per2 and Ferroptosis, Oxidative Medicine and Cellular Longevity. (2022) 2022, no. 1, 1–20, 10.1155/2022/8063897. PMC9588383 36285301

[98] Nan Y. , Su H. , and Lian X. , et al.Pathogenesis of Liver Fibrosis and Its TCM Therapeutic Perspectives, Evidence-Based Complementary and Alternative Medicine. (2022) 2022, 10.1155/2022/5325431, 5325431. 35529927 PMC9071861

[99] Roehlen N. , Crouchet E. , and Baumert T. F. , Liver Fibrosis: Mechanistic Concepts and Therapeutic Perspectives, Cells. (2020) 9, no. 4, 10.3390/cells9040875, 875. 32260126 PMC7226751

[100] Cao Y. , Yang H. , Huang Y. , Lu J. , Du H. , and Wang B. , Mesenchymal Stem Cell-Derived Exosomal miR-26a Induces Ferroptosis, Suppresses Hepatic Stellate Cell Activation, and Ameliorates Liver Fibrosis by Modulating SLC7A11, Open Medicine. (2024) 19, no. 1, 10.1515/med-2024-0945, 20240945. 38756248 PMC11097046

[101] Liu R. , Cui H. , and Li D. , et al.Roles and Mechanisms of Ferroptosis in Sorafenib Resistance for Hepatocellular Carcinoma, Journal of Hepatocellular Carcinoma. (2024) 11, 2493–2504, 10.2147/JHC.S500084. 39717509 PMC11665174

[102] Butt N.-U.-H. and Baytas S. N. , Advancements in Hepatocellular Carcinoma: Potential Preclinical Drugs and their Future, Current Pharmaceutical Design. (2023) 29, no. 1, 2–14, 10.2174/1381612829666221216114350. 36529919

[103] Tu S. , Zou Y. , and Yang M. , et al.Ferroptosis in Hepatocellular Carcinoma: Mechanisms and Therapeutic Implications, Biomedicine & Pharmacotherapy. (2025) 182, 10.1016/j.biopha.2024.117769, 117769. 39689515

[104] Yang M. , Xia L. , and Song J. , et al.Puerarin Ameliorates Metabolic Dysfunction-Associated Fatty Liver Disease by Inhibiting Ferroptosis and Inflammation, Lipids in Health and Disease. (2023) 22, no. 1, 10.1186/s12944-023-01969-y, 202. 38001459 PMC10668385

[105] Jiang Y. , Jiang K. , Sun P. , Liu Y. , and Nie H. , Oroxylin a Ameliorates Non-Alcoholic Fatty Liver Disease by Modulating Oxidative Stress and Ferroptosis Through the Nrf2 Pathway, Biochimica et Biophysica Acta (BBA) - Molecular and Cell Biology of Lipids. (2025) 1870, no. 5, 10.1016/j.bbalip.2025.159628, 159628. 40368273

[106] Chen S. , Xie J. , and Yin G. , et al.Hesperetin Alleviates High-Fat Diet-Induced NAFLD by Activating FXR to Modulate Ferroptosis Homeostasis and Suppress Inflammation, The Journal of Nutritional Biochemistry. (2026) 153, 10.1016/j.jnutbio.2026.110329, 110329. 41740765

[107] Xu H. , Sun J. , Ding N. , Zhang D. , and Li Y. , Targeting xCT-Mediated Amino Acid Metabolism: A Novel Mechanism of Silymarin in Ameliorating MAFLD-Associated Ferroptosis, The Journal of Nutritional Biochemistry. (2026) 152, 10.1016/j.jnutbio.2026.110304, 110304. 41654271

[108] Jiang T. , Xiao Y. , and Zhou J. , et al.Arbutin Alleviates Fatty Liver by Inhibiting Ferroptosis via FTO/SLC7A11 Pathway, Redox Biology. (2023) 68, 10.1016/j.redox.2023.102963, 102963. 37984229 PMC10694775

[109] Lou L. , Ye R. , and Huang Y. , Kaempferol Alleviates High-Fat-Induced Hepatocyte Injury by Inhibiting Ferroptosis, Tissue and Cell. (2026) 99, 10.1016/j.tice.2026.103323, 103323. 41544528

[110] Jiang Z. , Sun H. , and Miao J. , et al.The Natural Flavone Acacetin Protects Against High-Fat Diet-Induced Lipid Accumulation in the Liver via the Endoplasmic Reticulum Stress/Ferroptosis Pathway, Biochemical and Biophysical Research Communications. (2023) 640, 183–191, 10.1016/j.bbrc.2022.12.014. 36516527

[111] Ding S.-B. , Chu X.-L. , Jin Y.-X. , Jiang J.-J. , Zhao X. , and Yu M. , Epigallocatechin Gallate Alleviates High-Fat Diet-Induced Hepatic Lipotoxicity by Targeting Mitochondrial ROS-Mediated Ferroptosis, Frontiers in Pharmacology. (2023) 14, 10.3389/fphar.2023.1148814, 1148814. 37025486 PMC10070829

[112] Zhao M. , Mu H. , and Ji Q. , et al.Resveratrol Dual Efficacy in High-Altitude Hypoxia and NAFLD: Inhibits Ferroptosis by Modulating Key Proteins, including HIF-1α, ACSL4, and TfR1, Phytomedicine. (2026) 153, 10.1016/j.phymed.2026.157992, 157992. 41759426

[113] Choi J. , Choi H. , and Chung J. , Icariin Supplementation Suppresses the Markers of Ferroptosis and Attenuates the Progression of Nonalcoholic Steatohepatitis in Mice Fed a Methionine Choline-Deficient Diet, International Journal of Molecular Sciences. (2023) 24, no. 15, 10.3390/ijms241512510, 12510. 37569885 PMC10419585

[114] Zhu J. , Wu X. , Mu M. , Zhang Q. , and Zhao X. , TEC-Mediated tRF-31R9J Regulates Histone Lactylation and Acetylation by HDAC1 to Suppress Hepatocyte Ferroptosis and Improve Non-Alcoholic Steatohepatitis, Clinical Epigenetics. (2025) 17, no. 1, 10.1186/s13148-025-01813-3, 9. 39838504 PMC11748747

[115] Yi D. , Wu Z. , and Li X. , et al.Hydroxytyrosol Prevents Metabolic-Associated Steatohepatitis by Inhibiting SOCS2-Mediated Ferroptosis in High-Fat Diet Mice, Journal of Agricultural and Food Chemistry. (2026) 74, no. 7, 6265–6277, 10.1021/acs.jafc.5c16856. 41674361

[116] Li L. , Wang K. , and Jia R. , et al.Ferroportin-Dependent Ferroptosis Induced by Ellagic Acid Retards Liver Fibrosis by Impairing the SNARE Complexes Formation, Redox Biology. (2022) 56, 10.1016/j.redox.2022.102435, 102435. 36029649 PMC9425030

[117] Wang Y. , Kuang S. , and Li K. , et al.Astragalin Promotes HSCs Ferroptosis Through NCOA4 Mediated Ferritinophagy to Alleviate Liver Fibrosis in Zebrafish and Mice, Communications Biology. (2025) 8, no. 1, 10.1038/s42003-025-08421-0, 1081. 40691373 PMC12279947

[118] Li M. , Hu Y. , and Yu J. , et al.Baicalein Facilitates Hepatic Stellate Cell Ferroptosis via the DNMT1/SCARA5/GPX4 Axis, Phytomedicine. (2025) 149, 10.1016/j.phymed.2025.157554, 157554. 41270385

[119] Wang C. , Su Z. , Xu J.-H. , and Ko C.-Y. , Danshensu Attenuated Lipopolysaccharide-Induced LX -2 and T6 Cells Activation Through Regulation of Ferroptosis, Food Science & Nutrition. (2023) 11, no. 1, 344–349, 10.1002/fsn3.3065. 36655094 PMC9834887

[120] Liu G. , Wei C. , and Yuan S. , et al.Wogonoside Attenuates Liver Fibrosis by Triggering Hepatic Stellate Cell Ferroptosis Through SOCS1/P53/SLC7A11 Pathway, Phytotherapy Research. (2022) 36, no. 11, 4230–4243, 10.1002/ptr.7558. 35817562

[121] Yang Y. , Chen Y. , and Feng D. , et al. *Ficus hirta* Vahl. Ameliorates Liver Fibrosis by Triggering Hepatic Stellate Cell Ferroptosis Through GSH/GPX4 Pathway, Journal of Ethnopharmacology. (2024) 334, 10.1016/j.jep.2024.118557, 118557. 39009327

[122] Liu M. X. , Gu Y. Y. , and Nie W. Y. , et al.Formononetin Induces Ferroptosis in Activated Hepatic Stellate Cells to Attenuate Liver Fibrosis by Targeting NADPH Oxidase 4, Phytotherapy Research. (2024) 38, no. 12, 5988–6003, 10.1002/ptr.8338. 39475496

[123] Ying H. , Wang Y. , and Zhu D. , et al.Title: Resveratrol Ameliorates Liver Fibrosis by Inhibiting ATF4 to Regulate Glutamine Metabolism in Hepatic Stellate Cells, Archives of Pharmacal Research. (2025) 48, no. 11-12, 1420–1440, 10.1007/s12272-025-01586-6. 41258371

[124] Zhang D. , Tang Q. , and He X. , et al.Rubimaillin Ameliorates Liver Fibrosis by Triggering the Ferroptosis of Activated Hepatic Stellate Cells Through Targeting CPT1A, International Journal of Biological Sciences. (2026) 22, no. 4, 2065–2084, 10.7150/ijbs.120415. 41694586 PMC12905648

[125] Zhang J.-X. , Xiao Y. , and Li Y.-Q. , et al.Licochalcone a Induces Ferroptosis in Hepatocellular Carcinoma via Reactive Oxygen Species Activated by the SLC7A11/GPX4 Pathway, Integrative Cancer Therapies. (2023) 22, 10.1177/15347354231210867, 15347354231210867. 37965730 PMC10647947

[126] Xing Y. , Yang H. , Dai C. , Qiu Z. , Guan Y. , and Zhang L. , Investigating the Mechanism of Ferroptosis Induction by Sappanone A in Hepatocellular Carcinoma: NRF2/xCT/GPX4 Axis, European Journal of Pharmacology. (2024) 983, 10.1016/j.ejphar.2024.176965, 176965. 39214275

[127] Deng J. , Wu Z. , and Ning S. , et al.Curcumin Induces Ferroptosis in Hepatocellular Carcinoma by Regulating the P62-KEAP1-NRF2-Signaling Pathway, BMC Cancer. (2026) 26, no. 1, 10.1186/s12885-025-15307-1, 90. PMC12822296 41316098

[128] Ding Z. , Zheng M. , and Mou B. , et al.Emodin Induces Oxidative Stress and Ferroptosis in Hepatocellular Carcinoma Cells Through the miR-4465/NFE2L3/HMGCR/GPX4 Signaling Axis, Life Sciences. (2026) 388, 10.1016/j.lfs.2026.124200, 124200. 41534777

[129] Sun D. , Liu X. , and Mao C. , et al.Macelignan Targets SLC7A11 to Induce Ferroptosis and Modulate Macrophage Polarization via STAT6/PPAR-γ/KLF4 Signaling, Bioorganic Chemistry. (2026) 169, 10.1016/j.bioorg.2025.109422, 109422. 41456429

[130] Yang Y. , Chen J. , Gao Q. , Shan X. , Wang J. , and Lv Z. , Study on the Attenuated Effect of Ginkgolide B on Ferroptosis in High Fat Diet Induced Nonalcoholic Fatty Liver Disease, Toxicology. (2020) 445, 10.1016/j.tox.2020.152599, 152599. 32976958

[131] Zhang X. , Yin G. , and Chen S. , et al.Diosgenin Ameliorating Non-Alcoholic Fatty Liver Disease via *Nrf2* -Mediated Regulation of Oxidative Stress and Ferroptosis, Diabetes, Obesity and Metabolism. (2024) 26, no. 12, 5745–5756, 10.1111/dom.15945. 39344834

[132] Liu Y. , Zhou F. , and Zhao H. , et al.Dimeric Guaianolide Sesquiterpenoids From the Flowers of *Chrysanthemum indicum* Ameliorate Hepatic Steatosis Through Mitigating SIRT1-Mediated Lipid Accumulation and Ferroptosis, Journal of Advanced Research. (2025) 76, 345–370, 10.1016/j.jare.2024.12.047. 39788286 PMC12793765

[133] Liu W. , Li X. , Hu C. , Zhang H. , Wu Y. , and Hu Z. , Paris Saponin VII Inhibits Ferroptosis Through USP7/SLC7A11 to Alleviate Metabolic-Associated Fatty Liver Disease in Rats, Applied Biochemistry and Biotechnology. (2026) 198, no. 7, 5045–5066, 10.1007/s12010-026-05656-3. 41941048

[134] Yin X. , Liu Z. , Li C. , and Wang J. , Hinokitiol Ameliorates MASH in Mice by Therapeutic Targeting of Hepatic Nrf2 and Inhibiting Hepatocyte Ferroptosis, Phytomedicine. (2025) 139, 10.1016/j.phymed.2025.156472, 156472. 39922149

[135] Hu Y. , Lang Z. , and Li X. , et al.Ginsenoside Rg3 Promotes Hepatic Stellate Cell Ferroptosis by Epigenetically Regulating ACSL4 to Suppress Liver Fibrosis Progression, Phytomedicine. (2024) 124, 10.1016/j.phymed.2023.155289, 155289. 38176269

[136] Shen N. , Li M. , and Fang B. , et al.ALOX15-Driven Ferroptosis: The Key Target in Dihydrotanshinone I’s Epigenetic Battle in Hepatic Stellate Cells Against Liver Fibrosis, International Immunopharmacology. (2025) 146, 10.1016/j.intimp.2024.113827, 113827. 39675198

[137] Pang Q. , Zhou S. , and Wang Y. , et al.GAMG Alleviates Liver Fibrosis Through Inducing Ferroptosis in Inflammatory Macrophages via the IRF1/SLC7A11 Signaling Pathway, Redox Biology. (2025) 80, 10.1016/j.redox.2025.103509, 103509. 39904190 PMC11847116

[138] Lang Z. , Yu S. , and Hu Y. , et al.Ginsenoside Rh2 Promotes Hepatic Stellate Cell Ferroptosis and Inactivation via Regulation of IRF1-Inhibited SLC7A11, Phytomedicine. (2023) 118, 10.1016/j.phymed.2023.154950, 154950. 37441987

[139] Luo P. , Liu D. , and Zhang Q. , et al.Celastrol Induces Ferroptosis in Activated HSCs to Ameliorate Hepatic Fibrosis via Targeting Peroxiredoxins and HO-1, Acta Pharmaceutica Sinica B. (2022) 12, no. 5, 2300–2314, 10.1016/j.apsb.2021.12.007. 35646542 PMC9136576

[140] Lin L. , Li X. , Li Y. , Lang Z. , Li Y. , and Zheng J. , Ginsenoside Rb1 Induces Hepatic Stellate Cell Ferroptosis to Alleviate Liver Fibrosis via the BECN1/SLC7A11 Axis, Journal of Pharmaceutical Analysis. (2024) 14, no. 5, 10.1016/j.jpha.2023.11.009, 100902. 38784156 PMC11112007

[141] Lan T. , Wang W. , Zeng X.-X. , Tong Y.-H. , Mao Z.-J. , and Wang S.-W. , Saikosaponin a Triggers Cell Ferroptosis in Hepatocellular Carcinoma by Inducing Endoplasmic Reticulum Stress-Stimulated ATF3 Expression, Biochemical and Biophysical Research Communications. (2023) 674, 10–18, 10.1016/j.bbrc.2023.06.086. 37393639

[142] Ji J. , Cheng Z. , and Zhang J. , et al.Dihydroartemisinin Induces Ferroptosis of Hepatocellular Carcinoma via Inhibiting ATF4-xCT Pathway, Journal of Cellular and Molecular Medicine. (2024) 28, no. 8, 10.1111/jcmm.18335, e18335. 38652216 PMC11037408

[143] Sun Y. , Zhou Y. , and Huang D. , et al.Erk1/2 Orchestrates SSPH I-Induced Oxidative Stress, Mitochondrial Dysfunction and Ferroptosis in Hepatocellular Carcinoma, Journal of Cellular and Molecular Medicine. (2025) 29, no. 10, 10.1111/jcmm.70609, e70609. 40394754 PMC12092237

[144] Min L. , Wang H. , and Qi H. , Oridonin Induces Ferroptosis by Inhibiting Ubiquitin-Mediated Degradation of HMOX1 in Hepatocellular Carcinoma, Food and Chemical Toxicology. (2026) 209, 10.1016/j.fct.2025.115905, 115905. 41422936

[145] Yang R. , Gao W. , and Wang Z. , et al.Polyphyllin I Induced Ferroptosis to Suppress the Progression of Hepatocellular Carcinoma Through Activation of the Mitochondrial Dysfunction via Nrf2/HO-1/GPX4 Axis, Phytomedicine. (2024) 122, 10.1016/j.phymed.2023.155135, 155135. 37856990

[146] Shi B. , Li Y. P. , Gan Z. , and Chen P. , Monotropein Induced Ferroptosis to Alleviate the Progression of Hepatocellular Carcinoma via Regulating Nrf2/HO -1/GPX4 Axis, The Kaohsiung Journal of Medical Sciences. (2025) 41, no. 8, 10.1002/kjm2.70034, e70034. 40439491 PMC12407331

[147] Huang Q. , Li J. , and Ma M. , et al.High-Throughput Screening Identification of a Small-Molecule Compound That Induces Ferroptosis and Attenuates the Invasion and Migration of Hepatocellular Carcinoma Cells by Targeting the STAT3/GPX4 Axis, International Journal of Oncology. (2023) 62, no. 3, 10.3892/ijo.2023.5490, 42. 36825585 PMC9946807

[148] Jiang Y. , Ma P. , Yang Y. , Shi X. , Liu X. , and Sun M. , The Achilles’ Heel of Hepatocellular Carcinoma: Ginsenoside Compound K as a Novel GPX4 Degrader Promotes Ferroptosis in Hepatocellular Carcinoma, Journal of Translational Medicine. (2026) 24, no. 1, 10.1186/s12967-025-07587-9, 277. 41593603 PMC12918210

[149] Chen J. , Wang Z. , and Fu J. , et al.Ginsenoside Compound K Induces Ferroptosis via the FOXO Pathway in Liver Cancer Cells, BMC Complementary Medicine and Therapies. (2024) 24, no. 1, 10.1186/s12906-024-04471-9, 174. 38664638 PMC11044296

[150] LoBianco F. V. , Krager K. J. , and Johnson E. , et al.Parthenolide Induces Rapid Thiol Oxidation that Leads to Ferroptosis in Hepatocellular Carcinoma Cells, Frontiers in Toxicology. (2022) 4, 10.3389/ftox.2022.936149, 936149. 36591540 PMC9795200

[151] Zhang M. , Xu L. , and Zhu C. , et al.Magnoflorine Ameliorates Hepatic Fibrosis and Hepatic Stellate Cell Activation by Regulating Ferroptosis Signaling Pathway, Heliyon. (2024) 10, no. 22, 10.1016/j.heliyon.2024.e39892, e39892. 39634391 PMC11615489

[152] Yi J. , Wu S. , and Tan S. , et al.Berberine Alleviates Liver Fibrosis Through Inducing Ferrous Redox to Activate ROS-Mediated Hepatic Stellate Cells Ferroptosis, Cell Death Discovery. (2021) 7, no. 1, 10.1038/s41420-021-00768-7, 374. 34864819 PMC8643357

[153] Jin M. , Shi C. , Li T. , Wu Y. , Hu C. , and Huang G. , Solasonine Promotes Ferroptosis of Hepatoma Carcinoma Cells via Glutathione Peroxidase 4-Induced Destruction of the Glutathione Redox System, Biomedicine & Pharmacotherapy. (2020) 129, 10.1016/j.biopha.2020.110282, 110282. 32531676

[154] Zhu L. , Wu Y. , Chang S. , and Ci X. , Daphnetin Alleviates Liver Fibrosis by Inducing Ferritinophagy-Mediated Ferroptosis in Activated Hepatic Stellate Cells, Phytotherapy Research. (2026) 40, no. 2, 473–487, 10.1002/ptr.70134. 41351245

[155] Li D. , Li Y. , and Chen L. , et al.Natural Product Auraptene Targets SLC7A11 for Degradation and Induces Hepatocellular Carcinoma Ferroptosis, Antioxidants. (2024) 13, no. 8, 10.3390/antiox13081015, 1015. 39199259 PMC11351406

[156] Qu Z. , Zeng J. , Zeng L. , Li X. , and Zhang F. , Esculetin Triggers Ferroptosis via Inhibition of the Nrf2-xCT/GPx4 Axis in Hepatocellular Carcinoma, Chinese Journal of Natural Medicines. (2025) 23, no. 4, 443–456, 10.1016/S1875-5364(25)60853-3. 40274347

[157] Li J. , Wang Z. , Wei K. , Liu Q. , Liang L. , and Lin J. , Decursin Induces Ferroptosis via the NRF2/GPX4/SLC11A2 Axis and Suppresses Migration in Hepatocellular Carcinoma, Biomedicine & Pharmacotherapy. (2026) 195, 10.1016/j.biopha.2025.118941, 118941. 41496356

[158] Zahng Y. , Lan J. , Ma X. , Zhou Q. , Qin M. , and Gao L. , Exocarpium Citri Grandis Formula Granules Alleviate Fatty Liver Disease in Zebrafish by Maintaining Iron Homeostasis and Suppressing Lipid Peroxidation and Ferroptosis, Nan fang yi ke da xue xue bao = Journal of Southern Medical University. (2024) 44, no. 12, 2265–2275. 39725614 10.12122/j.issn.1673-4254.2024.12.01 PMC11683359

[159] Liu Y. , Ding X. , and Younas A. , et al.Xiaoyao San’s Dual Efficacy in NAFLD and Depression: Unraveling the Mechanisms via Network Pharmacology and Multi-Omics, Phytomedicine. (2025) 147, 10.1016/j.phymed.2025.157221, 157221. 40945251

[160] Cui L. , Lu M. , Li W. , Yin Y. , Li C. , and Chen M. , Hu Gan Tang Ameliorates Metabolic-Associated Fatty Liver Disease by Inhibiting Ferroptosis Through the Nrf2/GPX4 Pathway, Journal of Ethnopharmacology. (2025) 353, 10.1016/j.jep.2025.120342, 120342. 40752599

[161] Wang S. , Du R. , and Liu J. , et al.Multi-Approach Analysis Reveals the Mechanism by which Shugan Xiaozhi Decoction Protects Against Metabolic Dysfunction-Associated Steatohepatitis, Phytomedicine. (2025) 141, 10.1016/j.phymed.2025.156712, 156712. 40220418

[162] Fan X. , Gao J. , and Liu S. , et al.Integration of Network Pharmacology and Transcriptomics to Reveal the Mechanism of Paeoniae Radix Alba Polysaccharide in Alleviating Hepatic Fibrosis, Journal of Ethnopharmacology. (2025) 353, 10.1016/j.jep.2025.120357, 120357. 40759297

[163] Liu Y. , Li S. , Zhao X. , Tian J. , Xiao Z. , and Ma S. , The Effect and Mechanism of Albiflorin in Alleviating Hepatic Fibrosis by Mediating Hepatocyte Ferroptosis via the SLC7A11/GPX4 Signaling Pathway, Journal of Ethnopharmacology. (2026) 359, 10.1016/j.jep.2025.121080, 121080. 41429385

[164] Wang H. , Wang L. , Zhu F. , and Chen S. , Longchai Decoction Treated the Fibrosis of Liver Induced by CCl_4_ Regulates Nrf2/GPX4 Pathway to Suppress Ferroptosis, Journal of Cellular and Molecular Medicine. (2026) 30, no. 8, 10.1111/jcmm.71151, e71151. 42033213 PMC13109816

[165] Jiang X. , Yang J. , and Zhang Y. , et al.Yin-Dan-Ping-Gan Capsule Mitigates CCL4-Induced Liver Fibrosis via Regulating PPAR γ/GPX4 Signaling and Suppressing Ferroptosis, Pharmaceuticals. (2026) 19, no. 2, 10.3390/ph19020251, 251. 41754793 PMC12944426

[166] Xiao Z. , Gao S. , and Li S. , et al.Taohong Siwu Decoction Modulates Glutathione Metabolism to Suppress Hepatocyte Ferroptosis and Demonstrates Anti-Fibrotic Effects in the Liver, Journal of Ethnopharmacology. (2025) 350, 10.1016/j.jep.2025.120025, 120025. 40414577

[167] Hu J. , Zhang F. , and Qin X. , et al.Oxymatrine Inhibits Liver Cancer Progression by Regulating SIRT1/YY1/GPX4 Axis-Mediated Ferroptosis, Chemical Research in Toxicology. (2025) 38, no. 1, 46–57, 10.1021/acs.chemrestox.4c00208. 39729025

[168] Su B. , Wang S. , and Xiong Z. , et al.Hua Zheng San Ji Fang Promotes Ferroptosis Through Keap1/Nrf2 Pathway in Hepatocellular Carcinoma Cells, Journal of Ethnopharmacology. (2025) 352, 10.1016/j.jep.2025.120165, 120165. 40543691

[169] Han H. , Pei L. , and Dong Y. , et al.Plant Sterol Ester of α-Linolenic Acid Protects Against Ferroptosis in Metabolic Dysfunction-Associated Fatty Liver Disease via Activating the Nrf2 Signaling Pathway, The Journal of Nutritional Biochemistry. (2026) 147, 10.1016/j.jnutbio.2025.110098, 110098. 40915508

[170] Zhang J. , Zhang T. , Chen Y. , Xuan X. , Zhao Y. , and Lu G. , Spermidine Mitigates Ferroptosis in Free Fatty Acid-Induced AML-12 Cells Through the ATF4/SLC7A11/GCLM/GPX4 Pathway, Biochimica et Biophysica Acta (BBA) - Molecular and Cell Biology of Lipids. (2024) 1869, no. 8, 10.1016/j.bbalip.2024.159560, 110098. 39181440

[171] Huang P. , Zhu Y. , Qin Y. , Hu J. , Wang J. , and Qin J. , *Hypericum perforatum* L. Extract Alleviates Metabolic-Associated Fatty Liver Disease Through Inflammation, Lipid Metabolism and Ferroptosis Modulation: A Multi-Omics Perspective, Chinese Medicine. (2025) 20, no. 1, 10.1186/s13020-025-01248-1, 210. 41331663 PMC12673736

[172] Yu J. , Shao N. , and Yang J. , et al.Ultrafine Garlic Powder Alleviates Non-Alcoholic Steatohepatitis by Inhibiting Hepatocyte Ferroptosis and Modulating ERK-Dependent Oxidative Stress, Frontiers in Pharmacology. (2025) 16, 10.3389/fphar.2025.1711917, 1711917. 41347177 PMC12673841

[173] Sandech N. , Yang M. C. , and Juntranggoor P. , et al.Benja-Ummarit Induces Ferroptosis With Cell Ballooning Feature Through ROS and Iron-Dependent Pathway in Hepatocellular Carcinoma, Journal of Ethnopharmacology. (2024) 335, 10.1016/j.jep.2024.118672, 118672. 39127118

[174] Wang X. , Zhu W. , Xing M. , Zhu H. , Chen E. , and Zhou J. , Matrine Disrupts Nrf2/GPX4 Antioxidant System and Promotes Hepatocyte Ferroptosis, Chemico-Biological Interactions. (2023) 384, 10.1016/j.cbi.2023.110713, 110713. 37716422

[175] Xi C. , Zhou J. , Zheng X. , Fu X. , and Xie M. , Sodium Aescinate-Induced Hepatotoxicity via ATF4/GSH/GPX4 Axis-Mediated Ferroptosis, Scientific Reports. (2025) 15, no. 1, 10.1038/s41598-024-79723-2, 1141. 39774712 PMC11706965

[176] Huang C. , Jiang Y. , and Bao Q. , et al.Study on the Differential Hepatotoxicity of Raw Polygonum Multiflorum and Polygonum Multiflorum Praeparata and Its Mechanism, BMC Complementary Medicine and Therapies. (2024) 24, no. 1, 10.1186/s12906-024-04463-9, 161. 38632548 PMC11022370

[177] Xu S. , Chen Y. , and Miao J. , et al.Esculin Inhibits Hepatic Stellate Cell Activation and CCl4-Induced Liver Fibrosis by Activating the Nrf2/GPX4 Signaling Pathway, Phytomedicine. (2024) 128, 10.1016/j.phymed.2024.155465, 155465. 38471319

[178] Guo Q. , Wu Z. , and Wang K. , et al.Forsythiaside-a Improved Bile-Duct-Ligation-Induced Liver Fibrosis in Mice: The Involvement of Alleviating Mitochondrial Damage and Ferroptosis in Hepatocytes via Activating Nrf2, Free Radical Biology and Medicine. (2024) 222, 27–40, 10.1016/j.freeradbiomed.2024.05.042. 38815774

[179] Huang L. , Huang X.-H. , and Yang X. , et al.Novel Nano-Drug Delivery System for Natural Products and Their Application, Pharmacological Research. (2024) 201, 10.1016/j.phrs.2024.107100, 107100. 38341055

[180] Li Y.-Z. , Deng J. , and Zhang X.-D. , et al.Naringenin Enhances the Efficacy of Ferroptosis Inducers by Attenuating Aerobic Glycolysis by Activating the AMPK-PGC1α Signalling Axis in Liver Cancer, Heliyon. (2024) 10, 10.1016/j.heliyon.2024.e32288, e32288. 38912485 PMC11190665

[181] Han L. , Gao C. , and Jin X. , et al.Bioactive Natural Alkaloid 6−Methoxydihydrosanguinarine Exerts Anti−tumor Effects in Hepatocellular Carcinoma Cells via Ferroptosis, Frontiers in Pharmacology. (2025) 16, 10.3389/fphar.2025.1500461, 1500461. 40343005 PMC12058669

[182] Lai H.-C. , Weng J.-C. , and Huang H.-C. , et al.Solanum Torvum Induces Ferroptosis to Suppress Hepatocellular Carcinoma, Journal of Ethnopharmacology. (2024) 335, 10.1016/j.jep.2024.118670, 118670. 39117020

[183] Yang H. , Dai B. , and Chen L. , et al.Iberverin Downregulates GPX4 and SLC7A11 to Induce Ferroptotic Cell Death in Hepatocellular Carcinoma Cells, Biomolecules. (2024) 14, no. 11, 10.3390/biom14111407, 1407. 39595583 PMC11592392

